# Most dominant roles of insect gut bacteria: digestion, detoxification, or essential nutrient provision?

**DOI:** 10.1186/s40168-020-00823-y

**Published:** 2020-03-16

**Authors:** Tian-Zhong Jing, Feng-Hui Qi, Zhi-Ying Wang

**Affiliations:** 1 School of Forestry, Northeast Forestry University, Harbin, 150040 China; 2 School of Life Sciences, Northeast Forestry University, Harbin, 150040 China

**Keywords:** Poplar-and-willow borer, Anal secretion, Intestine bacterial community, Multiple factor analysis, Community pathway maps

## Abstract

**Background:**

The insect gut microbiota has been shown to contribute to the host’s digestion, detoxification, development, pathogen resistance, and physiology. However, there is poor information about the ranking of these roles. Most of these results were obtained with cultivable bacteria, whereas the bacterial physiology may be different between free-living and midgut-colonizing bacteria. In this study, we provided both proteomic and genomic evidence on the ranking of the roles of gut bacteria by investigating the anal droplets from a weevil, *Cryptorhynchus lapathi*.

**Results:**

The gut lumen and the anal droplets showed qualitatively and quantitatively different subsets of bacterial communities. The results of 16S rRNA sequencing showed that the gut lumen is dominated by Proteobacteria and Bacteroidetes, whereas the anal droplets are dominated by Proteobacteria. From the anal droplets, enzymes involved in 31 basic roles that belong to 7 super roles were identified by Q-TOF MS. The cooperation between the weevil and its gut bacteria was determined by reconstructing community pathway maps, which are defined in this study. A score was used to rank the gut bacterial roles. The results from the proteomic data indicate that the most dominant role of gut bacteria is amino acid biosynthesis, followed by protein digestion, energy metabolism, vitamin biosynthesis, lipid digestion, plant secondary metabolite (PSM) degradation, and carbohydrate digestion, while the order from the genomic data is amino acid biosynthesis, vitamin biosynthesis, lipid digestion, energy metabolism, protein digestion, PSM degradation, and carbohydrate digestion. The PCA results showed that the gut bacteria form functional groups from the point of view of either the basic role or super role, and the MFA results showed that there are functional variations among gut bacteria. In addition, the variations between the proteomic and genomic data, analyzed with the HMFA method from the point of view of either the bacterial community or individual bacterial species, are presented.

**Conclusion:**

The most dominant role of gut bacteria is essential nutrient provisioning, followed by digestion and detoxification. The weevil plays a pioneering role in diet digestion and mainly digests macromolecules into smaller molecules which are then mainly digested by gut bacteria.

## Background

The insect gut microbiota has been shown to contribute to the host’s digestion, detoxification, development, pathogen resistance, and physiology (reviewed in [1, 2]), as similarly documented in humans (reviewed in [3, 4]) and other animals (e.g., fish [5], chicken [6]). For example, cultivable bacteria isolated from the gut of *Dendroctonus rhizophagus* [7], *Antheraea assamensis*, *Helicoverpa armigera*, *Plutella xylostella* [8], and *Bombyx mori* [9], showing enzymatic capacity to hydrolyze cellulose, xylan, pectin, starch, lipids, and esters. An extracellular bacterium housed in specialized organs connected to the foregut helps a leaf beetle (*Cassida rubiginosa*) degrade pectin [10]. However, in vitro experiments can only be carried out with cultivable bacteria, and bacterial physiology may be different between free-living and midgut-colonizing [11].

Comparative studies with conventionally reared and aseptically reared insects showed that gut bacteria may contribute significantly to lipid digestion and protein digestion (mainly the latter), detoxification of secondary plant compounds, and modification of the volatile profiles of the insect host in *Anticarsia gemmatalis* [12], *Tenebrio molitor* [13], and also affecting survival, size, and egg production in mosquito [14]. A very recent study also showed that gut bacteria play an important role in insect insecticide resistance [15]. However, insects can digest some food and perform detoxification by themselves since genes encoding digestive and detoxifying enzymes are found in their genomes [16]. Thus, a question arises as to whether gut microbes enhance a certain enzyme activity or expand the digestion range (spectrum of substrates), which has not been adequately answered in previous studies. Furthermore, among these roles, there are no reports on which is the most dominant role for a given species.

For the past few years, metagenomic analysis has been extensively used for the study of the gut microbiome (reviewed in [17]). A very recent example showed that the gut microbiome of *P. xylostella* has thousands of genes from six families that encode carbohydrate-active enzymes [18]. However, this technique still cannot identify what portion of the gut microbiome is metabolically active and only gives indirect results inferred from microbial genomes [19] or metatranscriptomes [20]. Fortunately, metaproteomics or community proteogenomics has emerged to fill this gap (reviewed in [21]). Compared to that of metagenomics and metatranscriptomics, the major positive aspect of metaproteomics relies on “function” information. By identifying which proteins are observable and under what conditions, metaproteomics can reveal which community members are active and involved in specific biological processes under a particular ecological context [22].

Metaproteome measurements of gut microbiota are typically conducted with fecal samples, and proteins in the fecal samples need to be extracted by either a direct or indirect enrichment protocol [22]. In contrast to using fecal samples, this study demonstrated that proteomics analysis of insect anal droplets clearly shows the contributions of insects and gut microbes. The poplar-and-willow borer, *Cryptorhynchus lapathi* (L.) (Coleoptera: Curculionidae), is a wood-boring pest of economic importance throughout Europe, China, Japan, the USA, and Canada [23]. When disturbed, the larvae can produce anal droplets. The well-known honeydews are hemipteran honeydews that are responsible for plant wilt disease and have been investigated extensively. When disturbed, most species of carrion beetles defecate or produce anal defensive sprays [24–26]. Lepidopteran species, including tortricid (e.g., *Semutophila saccharopa* [27]) and some species of blue butterfly (e.g., *Polyommatus coridon* [28]), also excrete anal droplets. Previous studies have revealed the chemical composition of hemipteran anal droplets, which mainly contain sugar, amino acids, and other chemicals (reviewed in [29]). Proteins have also been detected in the anal droplets of *Nicrophorus* (burying beetles) [30] and aphids [29]. Furthermore, it has been demonstrated that the anal droplets of *C. lapathi* contain diverse proteins related to gut homeostasis [31]. Insect anal droplet enzymes and other proteins have three resources, namely, diet, secretion by gut epithelia or gut microbes, and sloughed epithelial cells and lysed microbes, providing a basis to investigate the interplay between host and gut microbes.

Using anal droplets as the study material has great advantages. First, we do not need to extract proteins or DNAs from guts or frasses. Second, we do not need to sacrifice animals and we can design repeated experiments to obtain longitudinal data. In this paper, the proteome of the larval anal droplet was investigated to assess gut bacteria functions on digestion, detoxification, and nutrient provisioning. The genomic data of gut bacteria were also investigated, yielding a similar result to that of the proteomic data. The variation between the two data sets was also determined.

## Results

### The bacterial community in the weevil gut

**Fig. 1:**
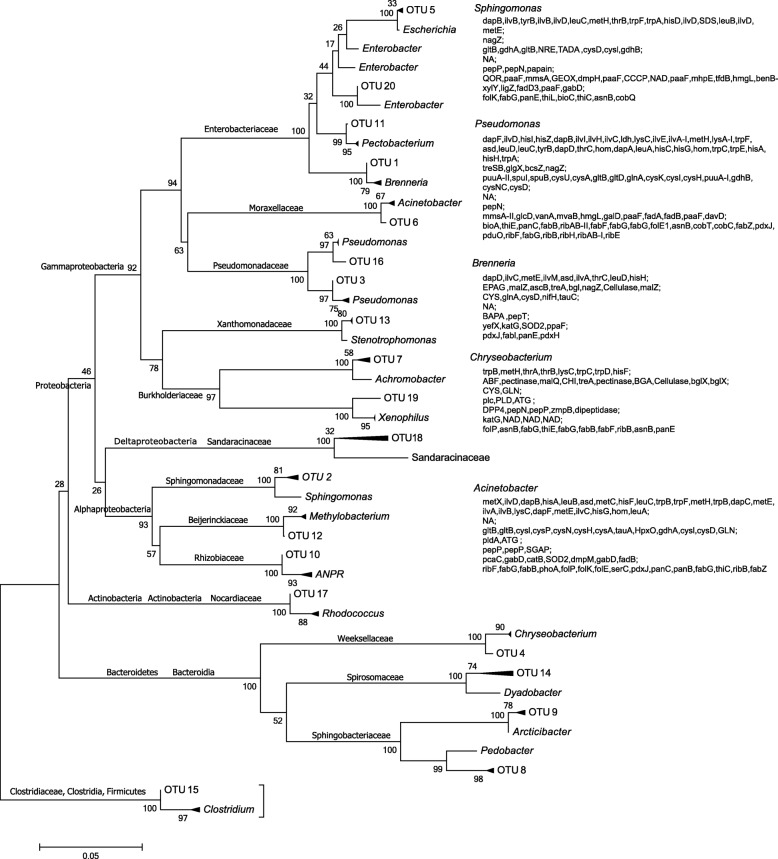
A phylogenetic tree of the top 20 sequences. The evolutionary history was inferred using the Neighbor-Joining method. The percentages of replicate trees in which the associated taxa clustered together in the bootstrap test (1000 replicates) are shown next to the branches. The evolutionary distances were computed using the Tamura 3-parameter method and are in the units of the number of base substitutions per site. The rate variation among sites was modeled with a gamma distribution (shape parameter = 5). The tree was collapsed into genus level. Proteins in gene names of the top five genera in RF of gut lumen sample are presented on the right side. The proteins are grouped into seven super roles and shown in seven rows separated by a semicolon. Vertically, the seven super roles are amino acid biosynthesis, carbohydrate digestion, energy metabolism, lipid digestion, protein digestion, PSM degradation, and vitamin biosynthesis. See Additional file 1: Table S5 for gene names of the proteins

Illumina paired-end sequencing yielded a total of 121,218 bacterial 16S ribosomal RNA (rRNA) gene sequences after trimming and quality control (Additional file 1: Table S1). Rarefaction analysis showed that neither curves reached the plateau phase, suggesting that the microbial communities were not sampled exhaustively (Additional file 2: Fig. S1). In total, 1665 operational taxonomic units (OTUs) (Additional file 1: Table S1) were identified by QIIME2, which were collapsed into 142 species (Additional file 1: Table S2) and 137 genera (Additional file 1: Table S3) after those with a frequency of less than two were excluded. The 20 most abundant OTUs belong to 13 families, 5 classes, and 4 phyla (Fig. 1).

**Fig. 2:**
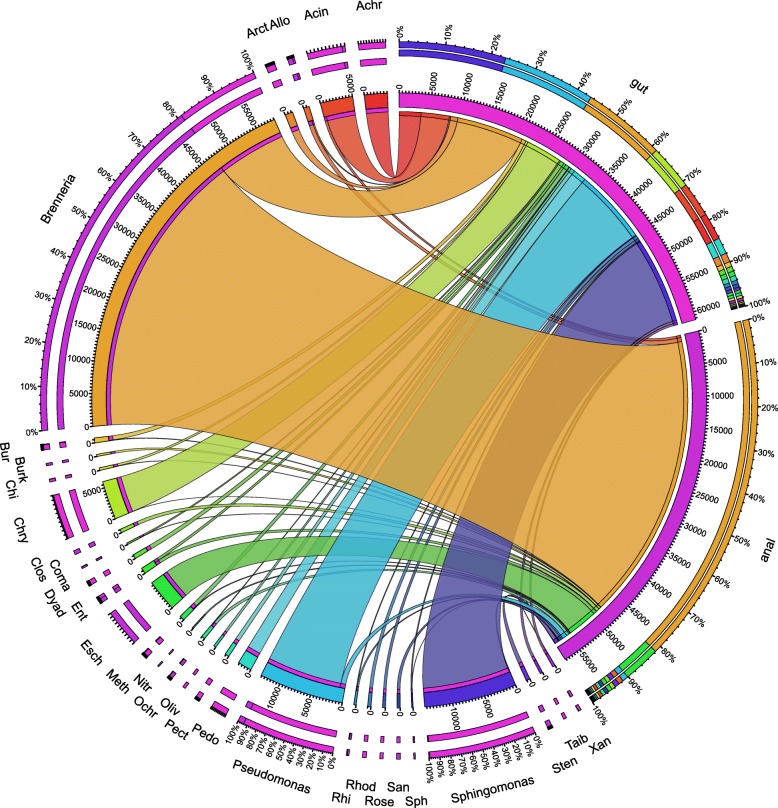
Bacterial genera identified from the guts and anal droplets. Only the top 20 in RF are shown. Chry, Chryseobacterium; Esch, Escherichia.Shigella; Acinr, Acinetobacter; Achr, Achromobacter; Pedo, Pedobacter; Arct, Arcticibacter; Ent, Enterobacteriaceae; Allo, Allorhizobium; Meth, Methylobacterium; Bur, Burkholderiaceae; Dyad, Dyadobacter; Pect, Pectobacterium; Sten, Stenotrophomonas; Ochr, Ochrobactrum; Clos, Clostridium; Rhod, Rhodococcus; San, Sandaracinaceae; Rose, Roseomonas; Xan, Xanthomonadaceae; Chi, Chitinophagaceae; Taib, Taibaiella; Oliv, Olivibacter; Rhi, Rhizobiaceae; Burk, Burkholderia; Coma, Comamonas; Sph, Sphingomonadaceae; Nitr, Nitrospira

The gut lumen and the anal droplets showed qualitatively and quantitatively different bacterial communities (Fig. 2, Additional file 2: Fig. S2, Additional file 1: Tables S3-4). The gut community was dominated by *Sphingomonas*, *Pseudomonas*, and *Brenneria*, of which the relative frequencies (RFs) are more than 15%, whereas the anal droplet community was dominated by *Brenneria* (~ 80% in RF). The Jaccard coefficient was 0.1746, indicating distinct communities on genus level. Pearson’s chi-squared test showed that there was a significant difference in genus abundance between the two groups (χ^2^ = 76045, df = 136, *p* value < 2.2e–16). At the phylum level, the gut lumen is dominated by Proteobacteria (> 80% in RF) and Bacteroidetes (> 18% in RF), with minor populations of Actinobacteria, Firmicutes, Deferribacteres, and Epsilonbacteraeota, the last two of which were not detected in anal droplets (Additional file 1: Table S4). In comparison with the gut lumen, the anal droplet is dominated by Proteobacteria (> 97% in RF), with minor populations of Firmicutes, Actinobacteria, Nitrospirae, Bacteroidetes, Patescibacteria, Deinococcus-Thermus, Gemmatimonadetes, Acidobacteria, Chloroflexi, Verrucomicrobia, Deferribacteres, and Epsilonbacteraeota, all of which were not detected in the gut lumen except Firmicutes, Actinobacteria, Bacteroidetes, Deferribacteres, and Epsilonbacteraeota (Additional file 1: Table S4). The Jaccard coefficient was 0.4167, indicating low similar communities on phylum level. Pearson’s chi-squared test showed that there was a significant difference in phylum abundance between the two groups (χ^2^ = 12252, df = 12, *p* value < 2.2e–16). Although the anal droplet bacterial community is more diverse in phyla, the gut lumen bacterial community has higher species diversity (3.63 vs 1.47 in Shannon index) (Additional file 2: Fig. S3) and higher species evenness (0.60 vs 0.28 in Pielou index).

### Proteins identified by MS

**Fig. 3:**
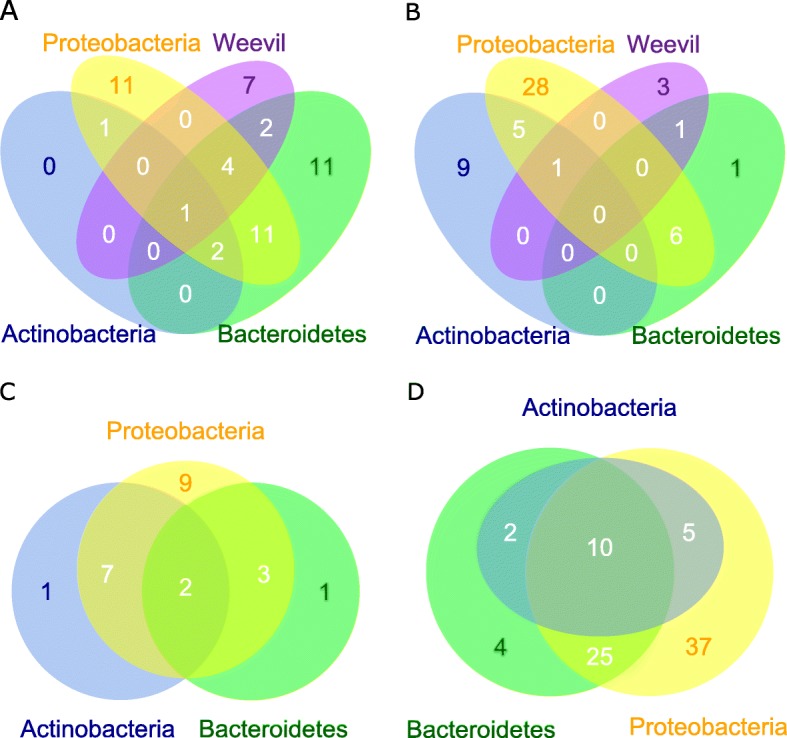
Venn plot of enzymes from the anal droplets. **a** Enzymes digesting diet nutrients. **b** Enzymes degrading PSM and xenobiotics. **c** Enzymes involved in nitrogen and sulfur metabolism. **d** Enzymes involved in the biosynthesis of essential amino acids and vitamins

The BLASTX results showed that 26,685 unigenes of *C. lapathi* were aligned to the proteome of *D. ponderosae*. The deduced amino acid sequences of these unigenes were used for database search by X!tandem. A total of 819 proteins derived from the weevil were identified. According to the results from the analysis of the gut lumen bacterial community (Additional file 1: Table S3), 13 pseudo-proteomes were chosen for bacterial protein identification (Table 1, Additional file 2: Fig. S4). In total, 707 proteins belonging to 210 enzymes were characterized as functioning dietary nutrient digestion, PSM and xenobiotic degradation, nitrogen and sulfur metabolism, and biosynthesis of essential amino acids (EAAs) and vitamins (Additional file 1: Tables S5-7). The proteins from the weevil were labeled by the UniProtKB entry names of their homologous genes of *Dendroctonus ponderosae* (Additional file 1: Table S7). Each origin contributes distinct enzymes (Additional file 1: Table S6), which were also summarized at the phylum level (Fig. 3). The enzyme overlap percentages among the bacteria range from 7.69 to 76.92%, with a median of 15.38% and a mean of 21.46%. The identified peptide sequences have been submitted to PASSEL with the identifier PASS01488.

**Table 1:** Bacterial species for pseudo-proteomic database establishment

Species	Organism code in KEGG and this study	Phylum	Identified protein count
*Achromobacter xylosoxidans*	axy	Proteobacteria	918
*Acinetobacter larvae*	ala	Proteobacteria	931
*Arcticibacter svalbardensis*	asv	Bacteroidetes	880
*Brenneria goodwinii*	bgj	Proteobacteria	984
*Chryseobacterium glaciei* IHB B 10212	chh	Bacteroidetes	866
*Dyadobacter fermentans*	dfe	Bacteroidetes	853
*Methylobacterium nodulans*	mno	Proteobacteria	906
*Pedobacter cryoconitis*	pcm	Bacteroidetes	856
*Pectobacterium carotovorum* subsp. *carotovorum* PC1	pct	Proteobacteria	971
*Pseudomonas putida* KT2440	ppu	Proteobacteria	1184
*Rhodococcus erythropolis* PR4	rer	Actinobacteria	961
*Roseomonas gilardii*	rgi	Proteobacteria	938
*Sphingomonas wittichii*	swi	Proteobacteria	972

### Diet digestive enzymes from the anal droplets

**Fig. 4:**
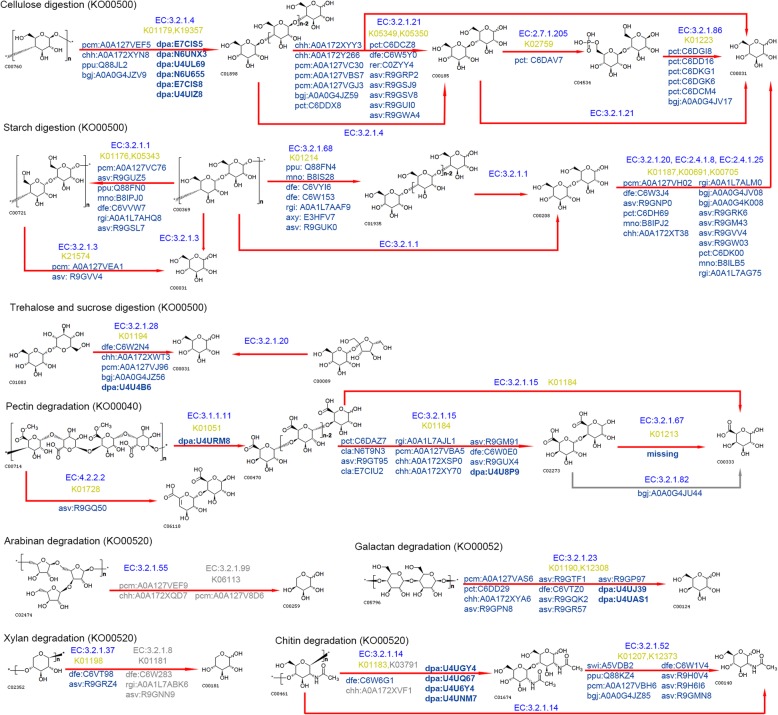
Droplet enzymes digesting carbohydrates. Enzymes are listed in entry IDs of UniprotKB database. Weevil-derived enzymes are shown in bold with their homologous proteins of *Dendroctonus ponderosae*. The enzymes in gray indicate they are not included in the current version of KEGG pathways

From the anal droplets, 113 proteins belonging to 20 enzymes that hydrolyze carbohydrates were identified (Additional file 1: Table S8). The community pathway maps based on these enzymes have been reconstructed (Fig. 4), showing the digestion routes of cellulose, starch, trehalose, sucrose, pectin, arabinan, galactan, xylan, chitin, etc. The maps also show the origins of the enzymes. The weevil-derived enzymes can only digest cellulose into cellodextrin or cellobiose, trehalose into glucose, pectin into pectate or digalacturonate, and chitin into chitobiose or GlcNAc, which can be further transformed into β-D-fructose 6-phosphate, joining glycolysis/gluconeogenesis, while other reactions are only catalyzed by bacteria-derived enzymes.

In total, 44 proteins belonging to 21 peptidases were identified from the anal droplets (Additional file 1: Table S9). The protein digestion community pathway maps are shown in Additional file 2: Fig. S5. The maps show that the weevil has both endopeptidases and exopeptidases, as do the bacteria. The weevil specifically secretes metallocarboxypeptidases, while the bacteria secrete distinct metalloendopeptidases, aminopeptidases, dipeptidases, dipeptidyl-peptidases and tripeptidyl-peptidases, and peptidyl-dipeptidases.

Enzymes that digest lipids were also identified from the anal droplets. As a result, 23 proteins belonging to 6 enzymes were identified (Additional file 1: Table S10). Community pathway maps for lipid digestion are shown in Additional file 2: Fig. S6. The maps show that gut bacteria and the host digest triacylglycerol into 1-acylglycerol or fatty acid and digest phosphatides (phosphatidylcholine, phosphatidylethanolamine, etc.) into lysophosphatides (lysolecithins, lysophosphatidylethanolamines, etc.), phosphatidates, or diacylglycerol. The weevil only contributes triacylglycerol lipase and lysophospholipase, while the bacteria contribute other enzymes alone.

### Anal droplet enzymes degrading plant secondary metabolites (PSMs) and other xenobiotics

In total, 115 proteins belonging to 56 enzymes that degrade PSMs and other xenobiotics were identified from the anal droplets (Additional file 1: Table S11). Community pathway maps are shown in Additional file 2: Figs S6-7. The maps indicate that both the weevil and bacteria contribute enzymes to the reactions converting lignin to lignin-derived biaryls. The weevil only contributes phenoloxidases (laccases, tyrosinases), whereas the bacteria contribute DyPs, MnSODs, and catalases. Lignin-derived biaryls can be degraded into pyruvate or succinyl-CoA by bacteria-derived enzymes, whereas weevil-derived enzymes catalyzing these reactions were not detected.

Enzymes that degrade other phenolics were only identified as the bacteria-derived enzymes. These enzymes can degrade 4-hydroxybenzoate, benzene, benzoate, and toluene into succinyl-CoA or pyruvate; 3-hydroxybenzoate, m-xylene, o-xylene, p-xylene, gallate, and 3-fluorobenzoate into pyruvate; 4-chlorophenol into cis-acetylacrylate, geraniol (Geranoyl-CoA) into 3-methylcrotonyl-CoA, and androstanedione into HIP-CoA or pyruvate.

Enzymes that degrade nonprotein amino acids were only identified as bacteria-derived enzymes. These enzymes can degrade beta-alanine into acetyl-CoA, taurine into l-cysteine, and GABA into succinate. We performed BLASTP against the sequence of the enzymes presented in ref. [32], and only bacterial MnSODs were identified as lignin-modifying enzymes (Additional file 1: Table S11, Additional file 2: Fig. S6). Neither bacterial glutathione-dependent β-etherases nor lignolytic dioxygenases were identified from the anal droplets. However, five weevil-derived P450s and one phenoloxidase (Additional file 1: Table S6) were identified from the anal droplets. One GST (HPGDS), one UGT, and five ABC transporters were also identified (Additional file 1: Table S6).

### Enzymes involved in nitrogen and sulfur metabolism

In total, 94 proteins belonging to 23 enzymes involved in nitrogen and sulfur metabolism were identified from the anal droplets (Additional file 1: Table S12). Community pathway maps (Additional file 2: Fig. S7) indicate that these enzymes transform urate or NH_3_ into l-glutamate, N_2_ or nitrate into NH_3_, taurine into l-cysteine, and sulfate into sulfide.

### Anal droplet enzymes involved in microbial EAA and vitamin biosynthesis

Bacterial enzymes involved in biosyntheses of EAAs and vitamins were identified from the anal droplets by module reconstruction (Additional file 1: Tables S12-13)). Community pathway maps (Additional file 2: Figs S8-9) indicate that these enzymes are all derived from gut bacteria. These enzymes synthesize histidine from PRPP, isoleucine from pyruvate, leucine from 2-oxoisovalerate, lysine from aspartate, methionine from aspartate, phenylalanine from chorismate, threonine from aspartate, tryptophan from chorismate, and valine from pyruvate. For vitamins, thiamine-P/thiamine-2P (VB1) is synthesized from AIR, riboflavin/FMN/FAD (VB2) from GTP, pyridoxal-5P (VB6) from erythrose-4P, pantothenate (VB5) from valine/l-aspartate, biotin (VB7) from malonyl-ACP, tetrahydrofolate (VB9) from GTP, and cobalamin (VB12) from cobinamide.

### Evaluation of bacterial roles

The percentages of catalyzed reactions (Fig. 4, Additional file 2: Figs S5-9) of each role for each bacterial species are shown in Additional file 1: Tables S14 and S15. Weighted by the abundance of each species, the percentages are summed as a score to indicate the magnitude of each role of the whole gut bacteria community. The scores from the proteomic data indicate that the most dominant role of gut bacteria is amino acid biosynthesis, followed by protein digestion, energy metabolism, vitamin biosynthesis, lipid digestion, PSM degradation, and carbohydrate digestion, while the order for the genomic data is amino acid biosynthesis, vitamin biosynthesis, lipid digestion, energy metabolism, protein digestion, PSM degradation, and carbohydrate digestion. The Spearman coefficient of the scores between the proteomic and genomic data is 0.68.

**Fig. 5:**
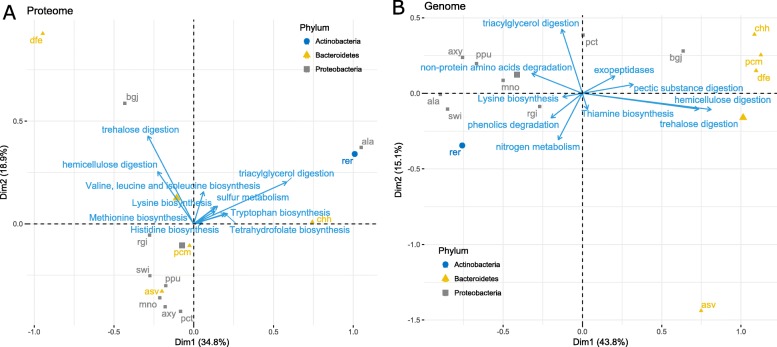
Biplots of bacterial species and roles from PCA analyses. **a** Proteomic data. **b** Genomic data. Only the top 10 roles on cos2 value are shown

Principal component analysis (PCA) was used to investigate whether the gut bacteria form functional groups at the basic role level. The PCA results from the proteomic data showed that the gut bacteria form four functional groups: chh; rer and ala; dfe and bgj; and the others (Fig. 5a). PC1 is characterized by triacylglycerol digestion, tryptophan biosynthesis, and tetrahydrofolate biosynthesis, while PC2 is characterized by trehalose digestion (cos2 > 0.6, Additional file 1: Table S16). The genomic data show four functional groups: asv; chh, pcm, and dfe; pct and bgj; and the others (Fig. 5b). PC1 is characterized by hemicellulose digestion, trehalose digestion, lysine biosynthesis, and pectic substance digestion, while PC2 is characterized by triacylglycerol digestion, thiamine biosynthesis, and nitrogen metabolism (cos2 > 0.6, Additional file 1: Table S17).

**Fig. 6:**
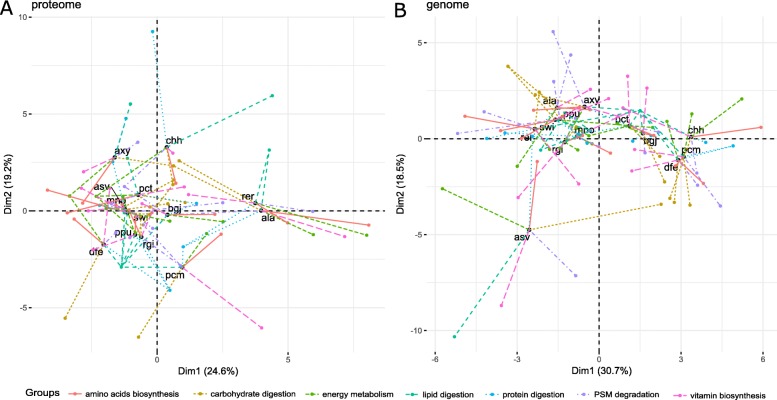
MFA analysis of proteome data (**a**) and genome data (**b**). Each function group is represented by a dot, and for each bacterial species, a line connects the species factor scores to the partial factor scores of a given group for this species. For a given individual, the point corresponds to the mean individual or the center of gravity of the partial points of the individual. That is, the individual viewed by all groups of variables

Multiple factor analysis (MFA) was used to compare super roles among bacterial species. Based on the proteomic data of the anal droplets, a graph of partial individuals was plotted (Fig. 6a). The results for individuals obtained from the analysis performed with a single group are considered the results of partial individuals. In other words, an individual considered from the point of view of a single group is called a partial individual. Figure 6 shows the functional profile of each bacterial species. In Fig. 6a, for example, rer and ala share a similar functional profile, e.g., similarly small on protein digestion, similarly moderate on vitamin biosynthesis, and similarly very small on carbohydrate digestion. Other partial species can be interpreted like this. From Fig. 6a, it appears that the gut bacteria form functional groups: pcm; chh; rer and ala; and the others. According to the loadings of each variable on each dimension (Additional file 1: Table S18), PC1 is characterized by amino acid biosynthesis, energy metabolism, and vitamin biosynthesis (cos2 > 0.2), PC2 is characterized by protein and lipid digestion (cos2 > 0.3), and PC3 is characterized by carbohydrate digestion and PSM degradation (cos2 > 0.15).

Based on the genomic data of the bacterial species identified from the gut of the weevil, a graph of partial individuals is also plotted (Fig. 6b). The bacteria can be classified into four groups: asv; chh, pcm, and dfe; pct and bgj; and the others. According to the loadings of each variable on each dimension (Additional file 1: Table S19), PC1 is characterized by amino acid biosynthesis, energy metabolism, protein, carbohydrate digestion, and PSM degradation (cos2 > 0.25), while PC2 is characterized by vitamin biosynthesis and lipid digestion (cos2 > 0.22).

**Fig. 7:**
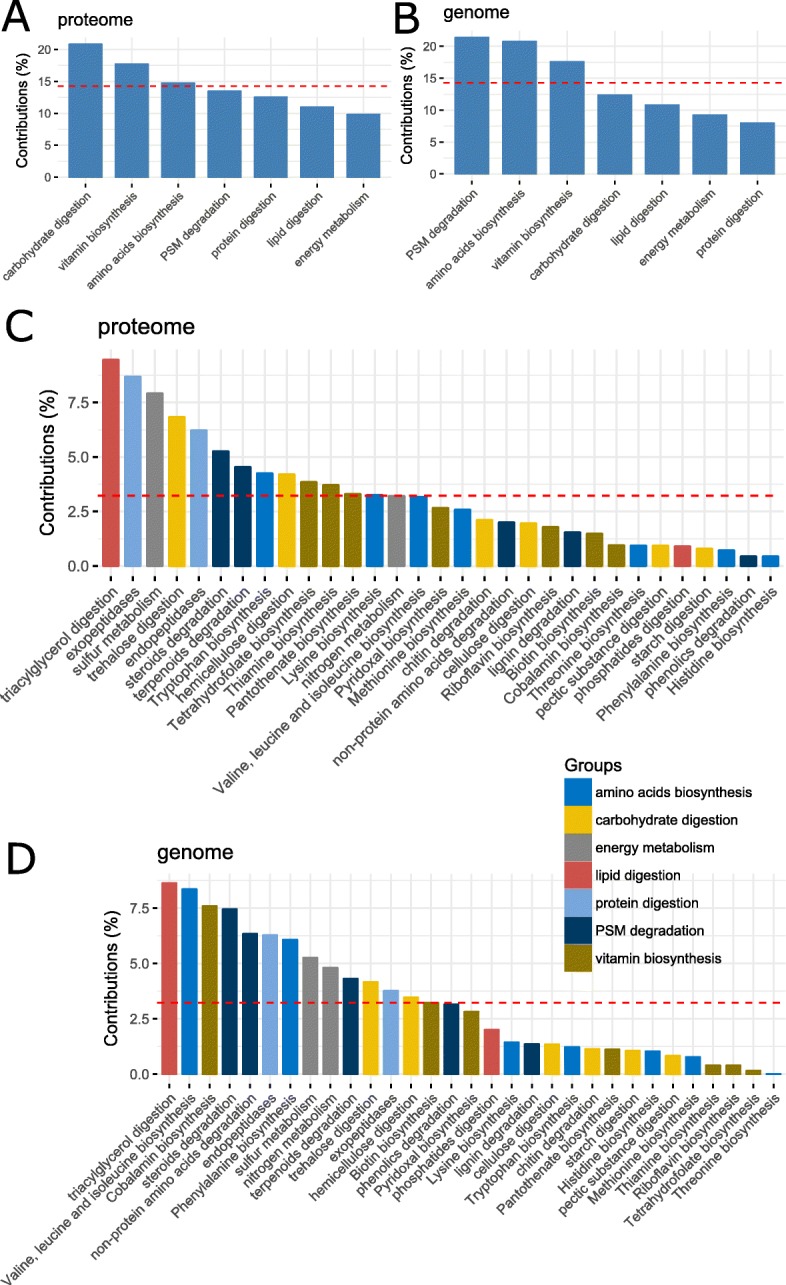
The contributions (in percentage) of variable groups (bacterial super roles) (**a**, **b**) and variables (bacterial basic roles) (**c**, **d**). The variability in principal components 1–7 accounted by **a**–**d** is 89.0%, 90.0%, 91.8%, and 91.0%, respectively. **a**, **c** Proteomic data. **b**, **d** Genomic data

The results from MFA also indicate the functional variations among gut bacteria (Fig. 7). The results based on the proteomic data showed that carbohydrate digestion has the top variation, followed by vitamin biosynthesis, amino acid biosynthesis, PSM degradation, protein digestion, lipid digestion, and energy metabolism (Fig. 7a), while the order for the genomic data is PSM degradation, amino acid biosynthesis, vitamin biosynthesis, carbohydrate digestion, lipid digestion, energy metabolism, and protein digestion (Fig. 7b). The variation in the basic role is presented in Fig. 7c, d. For proteomic data, triacylglycerol digestion has the highest variation whereas histidine biosynthesis has the lowest variation (Fig. 7c). For the genomic data, triacylglycerol digestion still has the highest variation while threonine biosynthesis has the lowest variation (Fig. 7d).

**Fig. 8:**
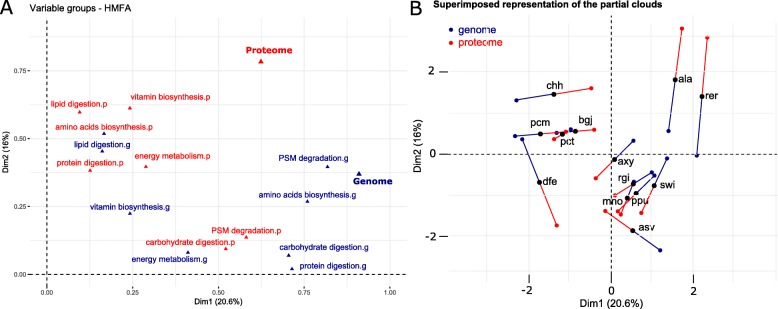
HMFA analysis on proteome and genome data. **a** Functional variation between the results from proteomic and genomic data. **b** Variation between proteomic and genomic data for each bacterial species

To compare the results from the proteomic and genomic data, a hierarchical multiple factorial analysis (HMFA) was carried out (Fig. 8). As shown in Fig. 8a, the counter diagonal partitions the functional groups into two parts: proteome (above triangle) and genome (lower triangle), which indicates clear variation between proteomic and genomic data except for lipid digestion, carbohydrate digestion, and PSM degradation. Thus, Dim1 is specific to the genomic point of view, while Dim2 is specific to the proteomic point of view. As shown in Fig. 8b, Dim1 and Dim2 partition the species into four groups. Species in the first and third quadrants have farther superposed representations, indicating farther variation between the proteomic and genomic data, whereas those species in the second and fourth quadrants have closer superposed representations, indicating closer variation between proteomic and genomic data.

## Discussion

In many insects, resident microbes often promote insect fitness by contributing to nutrition and detoxification [2]. To understand how gut bacteria function in vivo, we investigated the proteome of the anal droplets of a wood borer, *C*. *lapathi*, to reconstruct these biological pathways.

### Distinct microbiota between gut and anal droplets

We characterized the gut bacterial community of the larval weevil, although a diversity comparison of gut bacteria is not the focus of this study. Our results showed that the bacterial community of the gut is distinctive from that of the anal droplet (Additional file 1: Tables S2-4), and the gut has a more diverse bacterial community than the anal droplets (3.63 vs 1.47, respectively, Additional file 2: Fig. S3). Similar results have been presented in burying beetles *Nicrophorus defodiens* [33] and *Nicrophorus vespilloides* [34]. Therefore, the proteomic data of the gut bacteria were used for metaproteomics analysis. In our study, the gut bacterial community is dominated by Proteobacteria (86.68% in RF), which is in agreement with other studies based on stem-feeding species *Rhynchophorus ferrugineus* [35], wood-feeding coleopteran insects *Dendroctonus* spp. [36–40], and rice-feeding species *Lissorhoptrus oryzophilus* [41]. However, other reports have also documented Spirochaetes, Firmicutes, and Bacteroidetes to be dominant in termites [42, 43], of which gut microbiota have been studied extensively. Although similar abundance is observed at the phylum level, the microbiota at the genus level may be distinct. For example, the weevil in our study is dominated by *Sphingomonas*, *Pseudomonas*, and *Brenneria*, whereas *D. rhizophagus* is dominated by *Stenotrophomonas* and *Rahnella* [36], and *D. valens* is dominated by *Providencia* (35%) and *Enterobacter* (31%) [37]. The differences in insect gut bacterial diversity may be due to environmental habitat, diet, developmental stage, and phylogeny of the host [44].

### Proteomic and genomic analyses showing the ranking of the roles of the gut bacterial community

Using metaproteomics, we identified both weevil- and bacteria-derived enzymes from the anal droplets. Venn plots show that both the weevil and the gut bacteria have distinctive enzymes for both nutrient digestion and PSM degradation, suggesting a cooperative interaction between the weevil and its gut bacteria.

Bacteria often show incomplete metabolic pathways. For example, no complete pathways for the biosynthesis of biotin, pantothenate, pyridoxine, cobalamin, and menaquinone are present in any of the bifidobacterial genomes sequenced so far [45]. The investigation in our study showed similar results (Additional file 1: Table S15). There is no clear conclusion that a species lacks a function if it has an incomplete corresponding KEGG pathway in its genome; it may be true for a single species, but not for a gut microbial community. Let us take carbohydrate digestion as an example to explain this hypothesis. There are two main paradigms for diet digestion: the cellulosome system and polysaccharide utilization loci-like systems (PULs). In bacteria such as *Firmicutes*, degradative capacity is largely restricted to the cell surface and involves elaborate cellulosome complexes in specialized cellulolytic species. By contrast, in bacteria such as *Bacteroidetes*, utilization of soluble polysaccharides, encoded by PULs, entails outer membrane-binding proteins, and degradation is largely periplasmic or intracellular [46]. It is obvious that extracellular enzymes from different bacteria can catalyze similar/identical steps in a biodegradation route in the gut lumen. Given that the enzymes are fixed in the cellulosome complex, the bacteria flow, as do the substrates. For those intracellular reaction chains, for example, in the human gut, there are several pairs of organisms whose vitamin synthesis pathway patterns complement those of other organisms, suggesting that human gut bacteria actively exchange B vitamins among each other [47]. However, the cooperation may go further rather than stop at exchange of end products, and we hypothesized that even if none of the organisms had a complete enzyme system for a biosynthesis pathway (i.e., with only subpathways for each species), the community could perform a complete pathway provided that all subpathways can make up a complete pathway. In other words, gut microbes probably function in tandem by consuming the waste products of the others, leading to greater productivity of the microbial community and modifying the nutrients available to the host [48], which also indicates that interspecies interactions are essential for microbiota function [49]. If bacteria with some incomplete pathways could exchange “final products” (for example, vitamins) with others in the environment, why not “immediate products?” Burnum et al. (2011) reconstructed enzymatic pathways based on the proteins identified from the termite *Nasutitermes* P3 microbiome [50]. Intracellular and extracellular reactions in the gut bacteria of *Amitermes wheeleri* and *N. corniger* were also reconstructed based on gene/transcript abundance profiles [51]. Therefore, it is reasonable to reconstruct pathways/modules via KEGG based on identified gut proteins, which are called community or meta pathways/modules, for the reactions within a pathway/module do not necessarily all occur in one cell (Fig. 4; Additional file 2: Figs. S5-9). We also called the maps in Fig. 4 community pathway maps which are composed of ordinary KEGG pathway maps, enzymes’ organismal origin and accession numbers, and, if possible, other information—making the contribution of community members very clear on the maps.

This hypothesis also gives us an idea to rank the roles of the gut bacteria community. We calculated the percentage of reactions contributed by each species for each basic role or super role, and the percentages of all species were then summarized after weighted by the abundance of each species. We called this value the weighted sum functional score. The higher the functional score is, the more prominent the function is. The functional scores were calculated on either proteomic or genomic data. The results from the two data sets showed a similar functional order (0.68 Spearman coefficient). In light of these results, we conclude that the most dominant function of gut bacteria is essential nutrient provisioning, followed by digestion and detoxification.

Phylogenetic investigation of communities by reconstruction of unobserved states (PICRUSt) is a very popular software for predicting the functional composition of a metagenome based on 16S rRNA [52]. In this study, we did not use PICRUSt in a functional comparison between bacteria based on genomic data. PICRUSt uses KEGG Orthology (KO) to classify functions, whereas the basic role used in our study is a KEGG module under a KO number or a series of reactions that cross over several KOs. We did so because our goal is to rank roles within a species rather than quantify a certain role between treatments, as PICRUSt does. It is obvious that from Fig. 4 and Supplementary Figs. S5-9, a module or pathway comprises of distinctive reactions. To rank the roles within species, we need to permute these modules so that each basic role has a similar number of enzymes (represented by the EC number). Furthermore, the basic roles from genomic data are inferred according to proteomic data (i.e., Fig. 4 and supplementary Figs. S5-9), which makes our results more “metabolically active” than using a whole KO pathway. In principle, our approach shares the same idea with PICRUSt, i.e., using a product of species abundance and functional gene count to measure the bacterial community’s functional capabilities. However, our approach is not perfect and has some room for improvement. For example, the abundance of each species from the QIIME 2 results was used directly without considering the variation in 16S content between species. For the term “metabolically active,” it originally refers to metabolically active microbes within a microbiota [53]. Here, we describe metabolically active pathways/processes, which have extended the implication of this term.

As *C. lapathi* is a poplar-boring weevil, celluloses (~ 50%), hemicellulose (~ 20%), and lignin (~ 23%) are the primary components of its diet [54]. Poplar wood is low in protein (6–9 μg/mg dry weight (DW)) [55], and there are very few reports on the content of wood vitamins. Like other animals, insects lack the metabolic pathways for the synthesis of EAAs, as well as most vitamins [56]. By contrast, the weevil is able to digest nutrients and perform detoxification by its own enzymes (Fig. 4; Additional file 2: Figs S5-6). This fact may be the reason why the most dominant function of gut bacteria is essential nutrient provisioning rather than digestion or detoxification.

### Community pathway maps showing the metabolically active contribution of community members to diet digestion

Insect gut roles in digestion have been presented by two paradigms. One paradigm is that potential roles are extrapolated from the (meta) transcriptome [34, 51] or (meta) genome [51], while the other is that enzymatic roles are tested in vitro. However, most previous studies focused on separate species and never, to our knowledge, combined gut bacteria with their hosts. Therefore, so far, there is only limited knowledge about the cooperation in digestion either among gut bacteria or between bacteria and its host. In this study, we investigated the proteomes of an insect and its gut bacteria simultaneously and reconstructed community pathway maps to show the metabolically active cooperation between gut bacteria and the host, which helped to understand in depth the coevolution between the insect and its gut bacteria.

Our results showed that for cellulose digestion, both cellulase and β-glucosidases were detected from the anal droplets. Additionally, there are more weevil-derived cellulases than those from bacteria (6 vs 4, respectively, Additional file 1: Table S5), suggesting that the weevil itself plays a dominant role in the initial step of cellulose degradation. However, all β-glucosidases that degrade cellobiose produced by cellulase were identified as only being derived from bacteria. Furthermore, gut bacteria also supply cellobiose PTS permease and phosphocellobiase, which degrade cellobiose to cellobiose 6-phosphate and ultimately to glucose. This result is consistent with the proposal that the insect lacks exo-1,4-β-glucanases (cellobiohydrolases) [57]. In the KEGG pathway annotation of *D. ponderosae* (https://www.genome.jp/kegg-bin/show_pathway?dpa00500), the pathway of cellulose digestion is missing. On the community pathway maps shown in Fig. 4, we presented a complete community pathway from cellulose to glucose. Roles for each community member are clear on the maps, showing how cooperation is carried out by the weevil and its gut bacteria. Using metagenomic and metatranscriptomic analysis, the cellulose and hemicellulose degradation pathways were reconstructed in higher termites [51]. However, the information from either the host or the bacterial community members is missing.

The identified enzymes that digest amylose or hemicellulose were all bacteria-derived enzymes (Fig. 4). These results are consistent with those from genome sequencing studies that bacterial strains have several genes encoding enzymes involved in cellulose and hemicellulose degradation [58]. For digestion of pectin substances, pectin methylesterase (PME) was only identified as a weevil-derived enzyme, whereas α-l-arabinofuranosidases (ABFs) and α-1,5-arabinanases (ABNs) were bacteria-derived enzymes. In addition, ABNs are not included in the current version of KEGG pathway KO00520 (Fig. 4). In the wood-feeding higher termite *G. brachycerastes*, enzymes targeting hemicellulose and pectin have been identified by metagenomics; however, the authors did not provide a clear complementary route or any information from the host [35]. For the degradation of xylan to xylose, our study identified bacteria-derived β-xylanase, which is absent in the current KEGG pathway KO00520. There are very few studies on insect gut bacterial amylase. Nine gut bacterial isolates showing higher amylolytic activity have been isolated from the gut of the muga silkworm, *Antheraea assamensis*, by starch hydrolysis tests on starch agar plates [59]. Therefore, there has been no clear starch digestion route of the insect gut bacterial community prior to this study. In the current KEGG pathway of *D. ponderosae* (KO00500), the reaction from maltose to glucose is missing. By reconstruction of community pathway maps, a complete digestive pathway of starch is established, as shown in Fig. 4, indicating the contribution of each organismal origin.

Proteases and lipases have been detected in insect feces [60] and gut bacteria [59, 61], which have been paid little attention in previous studies on insect gut bacteria. In this study, the weevil contributes more endopeptidases and fewer exopeptidases than the bacteria (Additional file 2: Fig. S5). Triacylglycerol can be digested by both the weevil and the bacteria, whereas phosphatidylethanolamine and phosphatidylcholine digestion are mainly carried out by the bacteria (Additional file 2: Fig. S5). These results indicated that the weevil plays a pioneering role in diet digestion and mainly digests macromolecules into small molecules which are then mainly digested by gut bacteria.

In woody material, cellulolytic components of the plant cell wall are protected by lignin, representing a barrier for carbohydrate degradation in xylophagous insects [1]. Unfortunately, insects lack ligninases [57]. The degradation process of lignin in insect guts is poorly known. Extracellular enzymes involved in lignin degradation are lignin peroxidases (LiPs, ligninases), manganese peroxidases (MnPs, Mn-dependent peroxidases), versatile peroxidase (VPs), and dye-decolorizing peroxidases (DyPs), as well as laccases (benzenediol: oxygen oxidoreductase) [32, 62]. Two major classes of bacterial lignin-modifying enzymes are DyP-type peroxidases and laccases. Furthermore, several other bacterial enzymes, including glutathione-dependent β-etherases, MnSODs, katG, LigD, LigF, LigG, LigW/LigW2, LigY, LigX, LigZ, catechol dioxygenases, quinone oxidoreductase, acetyl-CoA acetyltransferase, enoyl-CoA hydratase, dehydrogenase, and cytochrome peroxidase, have recently been discovered and appear to play roles in lignin modifications [32, 63, 64]. Compared to bacteria, fungi, which are not covered in this study, are greatly known for their ability to depolymerize lignocellulosic biomass. In insect gut bacteria, Actinobacteria within the gut of a higher termite, *Amitermes hastatus*, have been shown to decolorize the dye RBBR and exhibit laccase and peroxidase activities [65], although the metagenome of the *Nasutitermes* P3 luminal community revealed no evidence for lignin degradation [66]. In termites, laccases produced by gut bacteria have been documented as lignin modification enzyme candidates [58]. In our study, however, gut phenoloxidases (POs) have been identified, of which the major function is demonstrated to be phenol-polymerization [67]. Therefore, the weevil may use its POs to degrade lignin. Furthermore, non-Actinobacteria bacteria-derived DyPs, catalases, quinine oxidoreductases, glycolate oxidases, cytochrome c peroxidases, and glutathione peroxidase were identified. These results indicate that gut bacteria may function predominantly in delignification [32]. However, efficient degradation of lignin does not appear to be necessary for lignocellulose degradation. Any structural modifications that improve the accessibility of polysaccharides to glycoside hydrolases will increase the efficiency of digestion. Mechanical grinding by insects will also increase the digestibility of lignocellulose, which cannot be ignored [68].

### Community pathway maps showing the metabolically active contribution of community members to the detoxification of PSMs/xenobiotics

Although the known PSMs are very large in number, the known PSM-degrading enzymes are few. The number of PSM-degrading enzymes detected from the anal droplet is also small. Nevertheless, our results showed that the weevil and gut bacteria contribute different enzymes (Fig. 3b). In contrast to PSM-degrading enzymes, a large number of xenobiotic-degrading enzymes were identified by KEGG annotation from the anal droplets, suggesting detoxifying roles for the gut bacteria. Gut microbes even correspond to the polystyrene degradation capacity in *Tenebrio molitor* [69]. The gut microbiota of pine weevil, *Hylobius abietis,* has the ability to degrade the diterpene acids of Norway spruce, and several genes of a diterpene degradation (*dit*) gene cluster were annotated via a metagenomic survey [70]. These results are consistent with those in humans and mammals where gut bacteria may play a major role in xenobiotic degradation (for review, see [71]). However, the degradation routes remain unclear. Bacterially facilitated insecticide resistance has been reported in the apple maggot *Rhagoletis pomonella* [72], bean bug *Riptortus pedestris* and allied stinkbugs [73], diamondback moth *Plutella xylostella* [15], oriental fruit fly *Bactrocera dorsalis* [74], and other insects [75], as deduced by the capacity of gut bacterial isolates to degrade insecticides. Genes encoding insecticide hydrolases have been identified based on comparative genomics analysis. However, these studies focused on a single isolate, and did not involve the degradation pathways of the gut bacterial community. Our results, demonstrated by the community pathway maps (Fig. S6), indicate that xenobiotics may be degraded by a wide variety of microorganisms, each of which degrades a small range of compounds. Unfortunately, our study only showed bacterial isolates with high frequency in conventionally colonized animals. Insecticide treatments are required to enrich insecticide-degrading isolates.

PSMs and xenobiotics may also stress gut microbes and change the host’s gut microbiota [76], and only those resistant to these compounds can survive. Compared with that in other figures, the number of organismal origins shown in Fig. S6 is sharply reduced, suggesting that only a tiny portion of gut bacteria detoxify xenobiotics. Thus, the weevil unites the gut microbes to detoxify PSMs and xenobiotics, which is mutually beneficial. From this perspective, gut microbes are mutualists rather than commensals.

Nonprotein amino acids (NPAAs) like γ-amino butyric acid (GABA), taurine, and β-alanine are abundant in the nervous systems of animals where they function in regulating neuronal excitability and thus behavior (reviewed in [77]). Feeding can potentially alter GABA concentrations in the insect nervous system and induce lethargic behavior, reduced growth, and reduced survival rates. As GABA levels usually increase rapidly in plants in response to insect attack (reviewed in [78]), it is vital for insects to degrade excessive amounts of APAAs. From the anal droplets, enzymes degrading β-alanine or GABA were identified as bacteria-derived enzymes, suggesting that gut bacteria play an important role in the homeostasis of insect NPAAs.

### Community pathway maps showing the metabolically active contribution of community members to the metabolism of nitrogen and sulfur

Many insects and other animals do not have the enzymatic capabilities required to produce ammonia from urate. In our study, community pathway maps of recycling nitrogenous wastes and fixing nitrogen were all identified as bacteria-derived pathways. Nitrogen recycling from stored uric acid in shield bugs and termites has been attributed to symbiotic microbes. Another hypothesis is that uric acid is transported to the gut, where it is catabolized by uricolytic microorganisms [79]. The bark beetles of the genus *Dendroctonus* feed on phloem, which is a nitrogen-limited source. Nitrogen fixation and nitrogen recycling may compensate for or alleviate such a limitation. It has been demonstrated that the P3 segment microbiome of higher termites is capable of fixing nitrogen [80], which has been confirmed by ^15^N_2_ incorporation in the gut of *Odontotaenius disjunctus*, a wood-feeding beetle native to the eastern USA [81]. The bacteria in the gut of *Dendroctonus rhizophagus* and *Dendroctonus valens* are able to recycle uric acid and contribute to insect N balance [82]. Based on the metaproteome, identical nitrogen fixation pathways to our study have also been established in higher termites, but missing information of the enzyme origins [50]. Compared with the termite, our study also presented routes for recycling nitrogenous wastes, which is another strategy to balance nitrogen levels. Our study provided additional concrete evidence that insects compensate for nitrogen deficiencies via gut bacteria, perhaps in a cooperative manner, which is helpful for deeply understanding nitrogen balance in insects.

Most insect species are unable to use reduce oxidized sulfur compounds and incorporate them into biomolecules, depending on their diet or the activity of their endosymbionts. The endosymbionts can assimilate sulfate into sulfur-containing amino acids such as Cys or Met [83, 84]. Our results showed that gut bacteria are able to transform taurine into l-cysteine and sulfate into sulfide, which indicated that gut bacteria help the weevil utilize sulfur compounds.

### Community pathway maps showing the metabolically active contribution of community members to essential nutrient supply

The gut microbiota has been shown to provide nutrients such as vitamins and EAAs to their hosts by functional assay with axenic animals [85–88] or by comparative functional analysis with PICRUSt [89]. Using community pathway maps, by contrast, our study provided possible pathways for vitamin and EAA biosynthesis by gut bacterial microbiota within the weevil (Additional file 2: Fig. S8-9) and the organismal origin of each reaction. Experimental deprivation of the microbiota of *Drosophila melanogaster* (axenic flies) revealed microbial sparing of dietary B vitamins and microbial promotion of protein nutrition [85]. Delayed development induced by toxicity can be partially prevented by vitamin B2 produced by gut bacteria [86]. The gut microbiota of *D. melanogaster* does provide thiamine to its host, enough to allow the development of flies on a thiamine-free diet [87]. δ^13^C stable isotope analyses revealed that gut microbes associated with Asian long-horned beetle *Anoplophora glabripennis* can serve as a source of EAAs when fed on nutrient-limited diets [88]. Using community pathway maps, our study showed that the gut bacterial community is capable of providing 9 EAAs for the weevil (Additional file 2: Fig. S8). Histidine biosynthesis is an unbranched pathway with ten enzymatic reactions, starting with phosphoribosyl pyrophosphate (PRPP) and leading to l-histidine [90]. The biosynthesis pathways of the three branched-chain amino acids (l-isoleucine, l-leucine, and l-valine) were reconstructed and were similar to those of any other bacteria [91]. There are multiple biosynthetic pathways in bacteria for the synthesis of lysine, including succinylase, dehydrogenase, and acetyllase [92], whereas only the succinylase pathway was reconstructed in this study. The biosynthesis pathways of methionine, tryptophan, threonine, and phenylalanine were reconstructed and were similar to those in *Escherichia coli* [93–96]. Our results also showed that the community pathways of the biosynthesis of VB1, VB11, and VB12 are identical to those in other extensively studied bacteria [97]. Bacteria synthesize pyridoxal 5′-phosphate (PLP) via two major pathways: a de novo pathway and a salvage pathway. There are two distinct de novo pathways in different organisms, either the deoxyxylulose 5-phosphate (DXP)-dependent or DXP-independent pathway [98]. In our study, the DXP-dependent pathway was reconstructed as a community VB6 biosynthesis pathway (Fig. S9), indicating that γ-proteobacteria play a dominant role in the gut community. The biosynthesis pathways of biotin pantothenate were reconstructed and were similar to those in the human gut microbiota [47]. For thiamine biosynthesis, only one branch of the whole pathway was reconstructed in this study. Proteobacteria, Bacteroidetes, and Actinobacteria contribute 40, 20, and 12 ECs for EAA biosynthesis, respectively, while the numbers of ECs for vitamin are 40, 23, and 5, respectively (Additional file 1: Table S13). These numbers suggest that Proteobacteria play a dominant role in the biosynthesis of EAAs and vitamins. However, antibiotic-treated insects do not suffer higher adult mortality than that in the control treatment [70], suggesting that the role of gut bacteria is no more than complementary for host survival.

### Gut bacteria function redundantly

It is proposed that every microbial taxon identified in an insect does not need to “have a function” [57]. Our results showed that the top 13 gut bacteria in terms of RF function redundantly. The PCA (Fig. 5) and MFA (Fig. 6) results indicated that gut bacteria clustered on the basic role level and super role level, respectively, regardless of the proteomic data or genomic data, which is consistent with the conclusion that the microbiomes of arthropods function as discrete groups [99]. Variation in microbiome traits is determined largely by environmental factors [100]. In this study, bacteria digesting cellulose and degrading lignin and PSMs were identified, which can also be considered to be the consequence of environmental selection. To study whether the microbiome of arthropods correlates with functional properties, single genes were associated to functional categories of either Cluster of Orthologous Genes (COG) or KEGG orthologous proteins by using predefined databases such as PRICUS (reviewed in [99]), or the metabolic traits associated with the bacterial taxa were examined [99]. In contrast to previous studies, we reconstructed pathways or modules with proteomic data and then calculated the percentages of enzymes contributed by each bacterial species out of all of the enzymes of each pathway or module, which is biologically more meaningful than the approach of previous studies. It is obvious that functional clusters from the genomic data couple as taxonomic clusters (Figs. 5b and 6b), whereas the results from proteomic data are not well coupled (Figs. 5a and 6a).

Our results also showed the variation between bacterial species on either the basic role or super role level (Fig. 7). The high contribution shown in Fig. 7 indicates high variation among bacterial species and vice versa. The higher the variation in a certain role is, the more important the part is that every bacterial species plays in this role. In other words, a high variation indicates that none of the bacterial species are redundant for a certain function, although there is no definite value for discrimination. However, this conclusion depends on a low overlap percentage of enzymes contributed by the bacteria. Our results showed that the median enzyme overlap percentage among bacteria is as low as 15.38% (Additional file 1: Table S6). These results suggest that the top 13 gut bacteria are necessary for the roles presented in this study. Although we did not present any results of enzymatic tests, previous studies have shown functional roles of isolates belong to the top 13 genera. For example, isolates of *Pseudomonas* or *Acinetobacter* from the gut of *D. rhizophagus* [7], *B. mori* [9], or *Saperda vestita* [101] showed amylolytic, cellulolytic, xylanolytic, lipolytic, and esterase activity. Insecticide-degrading *Pseudomonas*, *Sphingomonas*, and *Achromobacter* have repeatedly isolated from agricultural field soils, and these bacteria can be very quickly captured by insects as gut symbionts after insecticide application [102]. Insect gut-derived *Pseudomonas* capable of insecticidal degradation has been isolated from the apple maggot [72]. *Acinetobacter* isolate degrading phenol has been isolated from the gut of the termite [103]. These results provided concrete biological basis for this study.

## Conclusions

To understand how gut bacteria function in vivo, we first investigated the bacterial microbiota of the gut lumen and anal droplets from a wood borer, *C*. *lapathi*. A distinct bacterial community between the gut and the anal droplets was observed. The gut bacterial community structure is different from those of extensively studied wood-feeding higher termites. The proteome of the anal droplets of the weevil was investigated to rank the roles of the gut bacterial community. The most dominant role of the gut bacteria is essential nutrient provisioning, followed by digestion and detoxification. The results from genomic analyses of the gut bacteria showed a similar role order. The proteomic data were also used to reconstruct the community pathway maps showing the metabolically active portion of the gut bacterial community in the digestion of carbohydrates/proteins/lipids, detoxification of PSMs/xenobiotics, metabolisms of nitrogen/sulfur, and supply of essential amino acids/vitamins. The gut bacteria expand both the digestive and detoxifying spectrum of the weevil. The weevil probably plays a pioneering role in diet digestion and mainly digests macromolecules into smaller molecules, which are then mainly digested by gut bacteria. A possible cooperation mechanism of gut microbiota was also proposed in which members work in tandem to complete either extracellular or intracellular community pathways.

## Materials and methods

### Anal droplet collection

Larval weevils were collected in the wild near Harbin (N 46.00°, E 126.49°) in June. Anal droplets were collected directly onto foils placed against the anal areas of each weevil, while gently squeezing their abdomens. Samples on the foil were then transferred into a mini glass bottle with a pipette and stored at − 20 °C when not immediately used. Five larval weevils were used to collect anal droplets and were subsequently dissected to obtain the gut lumen. Each of the five anal droplet samples was split into two parts: part I was used for 16S rDNA amplification, and part II was used for Q-TOF MS analysis after being pooled with the other four part II droplet samples.

### Bacterial community profiling

The whole gut was dissected from the larvae on a clean bench and was put into a 1.5-mL Eppendorf tube with 1 mL of sterile ddH_2_O. The tube was shaken vigorously by a vortex to expose gut lumen. Then the five aqueous solutions containing gut lumen were used as templates for PCR using the 16S rRNA primers spanning the V3-V4 variable regions (PF: 5′-CCTACGGGAGGCAGCAG and PR: 5′-GGACTACHVGGGTWTCTAAT), and the DNA-free water was used as a template for the negative control. For each PCR, 25 μL of mixture was prepared, including 12.5 μL of Phusion® Hot Start Flex 2X Master Mix (New England Biolabs, Ipswich, MA, USA), 2.5 μL of each primer, 50 ng of template, and DNA-free water. The PCR involved a single denaturation step at 98 °C for 30 s, followed by 35 cycles of 98 °C for 10 s, 54 °C for 30 s, 72 °C for 45 s, and a final extension at 72 °C for 10 min. The five PCR products were pooled to construct a sequencing DNA library. The same process was performed on part I anal droplet samples. DNA libraries were validated by an Agilent 2100 Bioanalyzer (Agilent Technologies, Palo Alto, CA, USA) and quantified by a Qubit 2.0 Fluorometer. DNA libraries were multiplexed and loaded on an Illumina MiSeq instrument according to the manufacturer’s instructions (Illumina, San Diego, CA, USA). Sequencing was performed using a 2 × 300/250 paired-end (PE) configuration; image analysis and base calling were conducted by the MiSeq Control Software (MCS) embedded in the MiSeq instrument (see Additional file 3 for barcode sequences). Following sequencing, we used QIIME 2 (v2018.6.0) default parameters for quality filtering [104]. Replicated and chimeric sequences were removed using Vsearch implemented in QIIME 2 [105]. Sequences were clustered into OTUs at 97% sequence identity using cluster-features-de-novo implemented in QIIME 2 [105]. For each OTU, the most abundant sequence was chosen as a representative sequence, and taxonomic assignment was carried out with RDP classifier [106] using the SILVA database (release 132) [107]. An OTU table was generated in QIIME 2 (Additional file 1: Table S1). Rarefaction curves were plotted by subsampling the OTU table with step increments of 100 sequences and 100 iterations at each step. A genus-level heat map was generated using the R 3.4.3 package pheatmap. A phylogenetic tree of the top 20 sequences was reconstructed in the NJ tree which was built in MEGA6 [108]. The bacterial genera identified from the gut and anal droplets are shown in a figure drawn by Circos 0.69-6 [109].

### Q-TOF MS sequencing

Q-TOF MS sequencing was performed as described before [31]. The anal droplets were first separated by SDS-PAGE before LC-MS analysis. Ten microliters of pooled sample was resuspended in 50 μL of Laemmli sample buffer supplemented with 2% β-mercaptoethanol and heated at 95 °C for 5 min. After electrophoresis, the gel was rinsed with three changes of Nanopure water, stained for 20 min with Bio-safe TM Coomassie stain and destained with three changes of Nanopure water. The gel lane was carefully cut into eleven pieces (Additional file 2: Fig. S4), placed into Eppendorf tubes, and rinsed twice for 10 min with 1 mL of MilliQ water. After destaining with freshly prepared destaining solution (25 mM (NH_4_)HCO_3_, 50% acetonitrile), the gel pieces were dehydrated until they shrank and became white (approximately 2 min) with 25 mM (NH_4_)HCO_3_ with 50% acetonitrile and then once more for 30 s in 100% acetonitrile. The gel pieces were then rehydrated in freshly prepared 10 mM dithiothreitol for 1 h at 56 °C (water bath) and were alkylated with freshly prepared 55 mM iodoacetamide for 1 h at room temperature in the dark. Subsequently, the gel pieces were washed with 25 mM (NH_4_)HCO_3_ twice for 10 min and destained to become white as before. Trypsin digestion was performed overnight with trypsin working solution (1 μg/μL stock solution was diluted 15-fold with 25 mM (NH_4_)HCO_3_) at 37 °C. Digested proteins were extracted 4 times with 50 μL of 50 mM (NH_4_)HCO_3_, 50 μL of 0.1% (v/v) FA in water, 50 μL of 0.1% (v/v) FA in acetonitrile, and 50 μL of acetonitrile. All extracts were pooled, freeze-dried at − 20 °C, and resuspended in 0.1% FA for sequencing.

The resuspended peptides were fractionated using reversed-phase high-pressure liquid chromatography (HPLC; Prominence nano 2D, Shimadzu, Kyoto, Japan), and the gradient-eluted peptides were analyzed using a MicrOTOF-QII system (Bruker Daltonics, Billerica, MA, USA). The liquid chromatography columns were packed in-house with C18 (5 μm, 150 Å; Downers Grove, IL, USA). The LC-MS conditions were as follow: mobile phase: (a) 100% H_2_O with 0.1% FA and (b) 100% acetonitrile with 0.1% formic acid; gradient: 0–4 min, 5–5% B; 4–30 min, 5–40% B; 30–35 min, 40–80% B; 35–45 min, 80–80% B; 45–45.1 min, 80–5% B; 45.1–60 min, 5–5%; flow rate, 400 nL/min; drying gas temperature, 150 °C; capillary voltage, 1.5 kV; collision gas, argon. The results were exported as a .MGF file for X!tandem [110] analysis.

### Database searching and protein identification

For protein identification, the peak list data from MS were searched against a protein database. In a classic metaproteomics analysis, it is difficult to ascribe the species origin of identified proteins based on a closely related metagenome [22]. In this study, we constructed a protein database of *C. lapathi* itself to identify weevil-derived proteins [31], and used “pseudo-proteomes” for bacteria-derived proteins. First, a transcriptomic database of *C. lapathi* was constructed by de novo assembly (Trinity software (v r20140717) [111]) of the sequences from an Illumina sequencing platform (Illumina HiSeq2500) based on pooled RNAs of the larvae, pupae, and adults and was then clustered by CD-HIT software (v 4.5.4) (http://weizhongli-lab.org) to obtain unigenes. The unigenes were subsequently mapped to the proteome of *Dendroctonus ponderosae* with a cut-off E-value of 10^−4^ using BLASTX (v2.3.0) to obtain a proteomic database of *C. lapathi*.

For identification of bacterial proteins from the anal droplet, the peak list data from MS were also applied to search against proteomes of 13 pseudo-proteomes corresponding to the top 14 genera (the 10th genus was excluded because it only has family information) which accounted for 93.22% of the relative frequency of the gut bacteria community. The proteomes of *D. ponderosae* and bacteria were downloaded from UniProtKB (http://www.uniprot.org/). The database searches were performed by the R package, rTANDEM [110]. The proteins were identified from at least one peptide and with an X!tandem [112] score corresponding to an expected value of better than 0.05.

### Assignment of proteins to Gene Ontology (GO) Terms and Kyoto Encyclopedia of Genes and Genomes (KEGG) Pathways

The IDs of the weevil proteins identified by X!tandem were labeled with their homolog gene IDs (UniProtKB protein entry, http://www.uniprot.org) of *D. ponderosae*, and then, GO/KEGG IDs and EC numbers were obtained from UniProtKB using ID Mapping function. The modules and pathways were reconstructed by the KEGG Mapper function (https://www.genome.jp/kegg/mapper.html). Proteins were also annotated by local BLASTPs (v2.3.0) against sequences referring to the literature and/or NCBI conserved domain search (https://www.ncbi.nlm.nih.gov/Structure/cdd/wrpsb.cgi). For details, see Additional file 2.

### Data statistics

The EC numbers were used to identify an enzyme, and the GO numbers, KEGG numbers, and enzyme names were also used if the EC numbers were not available or if an enzyme had several EC numbers. Venn plotting was carried out to show the origin specification of the detected enzymes. The roles of gut bacteria were assigned into seven super roles and 31 basic roles (see the “Results” section). For example, pectin degradation, arabinan degradation, and galactan degradation were assigned as a basic role, pectic substance digestion. And cellulose digestion, chitin degradation, hemicellulose digestion, pectic substance digestion, starch digestion, and trehalose digestion were assigned to a super role, carbohydrate digestion. We hypothesized that the contribution of a bacterial species depends on how many reactions it catalyzed in a biological pathway. Therefore, we calculated the percentage of catalyzed reactions in each role for each bacterial species, which was subsequently used for principal component analysis (PCA), multiple factor analysis (MFA), and hierarchical multiple factorial analysis (HMFA). MFA proceeds in two steps: First, it computes a PCA of each data table and “normalizes” each data table by dividing all its elements by the first singular value obtained from its PCA. Second, all the normalized data tables are aggregated into a grand data table that is analyzed via a (non-normalized) PCA that gives a set of factor scores for the observations and loadings for the variables [113]. The PCA was performed to investigate whether gut bacteria clustered at the basic role level, the MFA was carried out to investigate whether gut bacteria clustered at super role level and to investigate the variations among super roles, and the HMFA was performed to compare the variations between the proteomic and genomic data. These analyses were performed using the R (v3.4.3) packages VennDiagram, FactoMineR, and factoextra. R codes have been shared at https://github.com/Jingtz/R-codes-for-microbiome-2019/tree/master.

## Supplementary information

**Additional file 1.** Supplementary tables S1-S19.

**Additional file 2.** Supplementary methods and Supplementary figures S1-S9.

**Additional file 3.** barcode sequences.

## Data Availability

All data generated or analyzed during this study are included in this published article [and its Additional information files].

## Abbreviations

ABFs: α-l-arabinofuranosidases
ABNs: α-1,5-arabinanases
ala: *Acinetobacter larvae*
asv: *Arcticibacter svalbardensis*
axy: *Achromobacter xylosoxidans*
bgj: *Brenneria goodwinii*
chh: *Chryseobacterium glaciei* IHB B 10212
cla: *Cryptorhynchus lapathi*
COEs: Carboxylesterases
dfe: *Dyadobacter fermentans*
DyPs: Dye-decolorizing peroxidases
GO: Gene Ontology
GSTs: Glutathione-S-transferases
HMFA: Hierarchical multiple factorial analysis
katG: Catalase-peroxidases
KEGG: Kyoto Encyclopedia of Genes and Genomes
LiPs: Ligninases
MFA: Multiple factor analysis
mno: *Methylobacterium nodulans*
MnPs: Mn-dependent peroxidases
MnSODs: Manganese-dependent superoxide dis-mutases
PCA: Principal component analysis
pcm: *Pedobacter cryoconitis*
pct: *Pectobacterium carotovorum* subsp. *carotovorum* PC1
PG: Polygalacturonase
PL: Pectin/pectate lyase
PME: Pectin methylesterase
ppu: *Pseudomonas putida* KT2440
PSM: Plant secondary metabolite
rer: *Rhodococcus erythropolis* PR4
RF: Relative frequencies
rgi: *Roseomonas gilardii*
swi: *Sphingomonas wittichii*
UGTs: UDP-glucuronlytransferases
VPs: Versatile peroxidase
