# Supplementary material for: Most dominant roles of insect gut bacteria: digestion, detoxification, or essential nutrient provision?
Source: Microbiome. 2020 Mar 16;8:38. doi: 10.1186/s40168-020-00823-y (PMC7077154; doi:10.1186/s40168-020-00823-y)
Supplement: Supplementary file 2 — Additional file 2. Supplementary methods and Supplementary figures S1-S9. [file 40168_2020_823_MOESM2_ESM.pdf]

Additional file 2

Identification of diet digestive enzymes

Enzymes digesting carbohydrates, proteins or lipids were identified according to the annotations from UniProtKB and KEGG, and/or referring to literatures after performing a local BLASTP (v2.3.0) and/or NCBI conserved domain search (https://www.ncbi.nlm.nih.gov/Structure/cdd/wrpsb.cgi). Traditionally, cellulose was thought to be degraded by three main types of enzyme activity: 1) cellulases (EC 3.2.1.4), 2) glucan 1,4-β-glucosidas (EC 3.2.1.74), cellulose 1,4-β-cellobiosidase (non-reducing end) (EC 3.2.1.91), cellulose 1,4-β-cellobiosidase (reducing end)(EC 3.2.1.176) and 3) β-glucosidases (EC 3.2.1.21)[5]. Xylanolytic enzymes includes α-L-arabinofuranosidase (EC:3.2.1.55), acetylxylan esterase (EC 3.1.1.72), and α-D-glucuronidase (EC:3.2.1.139) [13]. Oatspelt xylan can be degraded by α-L-arabinofuranosidase, xylanase (EC:3.2.1.8) and β-xylosidase (EC:3.2.1.37) in turn [15]. Among the pectinolytic enzymes, pectin methylesterase (PME) (EC 3.1.1.11) catalyzes the removal of methyl groups, improving the affinity of the pectin depolymerases pectin/pectate lyase (PL) and polygalacturonase (PG) for the pectin main chain. PL (EC 4.2.2.) uses a trans-elimination reaction for cleavage, thereby releasing unsaturated sugars, and PG employs a hydrolytic reaction for saturated sugars release. Among PGs, the endo-PG (EC 3.2.1.15) randomly hydrolyze internal sites of the pectin main chain, while the exo-PG promote a sequential cleavage from non-reducing ends liberating di (EC 3.2.1.82) or monogalacturonic acid residues (EC 3.2.1.67) (reviewed in [7]). The degradation of l-arabinan involves the synergistic activity of two major enzymes: α-l-arabinofuranosidases (ABFs; EC 3.2.1.55) and α-1,5-arabinanases (ABNs; EC 3.2.1.99) [19]. Dietary protein digesting enzymes includes

endopeptidases and exopeptidases. We also included dipeptidyl peptidase 4 (DPP4) and peptidase M4 family in this study. DPP4 has been shown an important digestive function in *Tenebrio molitor* larvae [18]. Peptidase M4 family includes several endopeptidases such as thermolysin (EC 3.4.24.27), aureolysin (the extracellular metalloproteinase from *Staphylococcus aureus*), neutral protease from *Bacillus cereus* and protealysin. Most of these secreted proteases degrade extracellular proteins and peptides for bacterial nutrition, especially prior to sporulation [12].

Identification of lignin-degrading enzymes

There are very few studies on insect lignin-degrading enzymes. Extracellular enzymes involved in lignin degradation are lignin peroxidases (LiPs, ligninases, EC 1.11.1.14), manganese peroxidases (MnPs, Mn-dependent peroxidases, EC 1.11.1.13), Versatile peroxidase (VPs, EC 1.11.1.16) and dye-decolorizing peroxidases (DyPs, EC 1.11.1.19), as well as laccases (benzenediol: oxygen oxidoreductase, EC1.10.3.2) [4, 9]. Two major classes of bacterial lignin-modifying enzymes are DyP-type peroxidases and laccases. Furthermore, recently also several other bacterial enzymes, Glutathione-dependent β-etherases, manganese-dependent superoxide dis-mutases (MnSODs), Catalase-peroxidases (katG, EC 1.11.1.21), three different types of enzymes involved in degradation of guaiacylglycerol-β-guaiacyl (LigD (a Cα-dehydrogenase), LigF (a β-etherase) and LigG (a glutathionelyase)) and four different types of enzymes involved in the initial steps of degrading a biphenyl compound (2,2’-dihydroxy-3,3’-dimethoxy-5,5’-dicarboxybiphenyl) (LigW/LigW2 (decarboxylases), LigY (a C-C hydrolase), LigX (an iron-dependent demethylase) and LigZ (an extradiol dioxygenase)), have been discovered

that seem to play a role in lignin modifications[4]. In addition, catechol dioxygenases are further classified as being “extradiol” or “intradiol,” both degrading lignin[16]. The role of NADPH: quinone oxidoreductase in degradation and depolymerization of lignin is well established and reported, and the expression of quinone oxidoreductase, acetyl-CoA acetyltransferase, enoyl-CoA hydratase, dehydrogenase (responsible for cleavage of ether linkage), and cytochrome peroxidase was expressed on lignin (reviewed in [11]).

For ligninlytic enzymes identification, protein sequences of DyP, laccase, LiP, MnP, VP and those presented in ref [3, 4], retrieved from NCBI, were used to setup local database for BLASTP and the results were confirmed by both Uniprot and NCBI CDD annotations, and for MnSOD identification, SWISS-MODEL (https://www.swissmodel.expasy.org/interactive) was also used. We did BLASTP against Peroxibase (http://peroxibase.toulouse.inra.fr/) for ligninlytic peroxidases from the anal droplet. In Genbank, ligXs (BAK65452, BAA36168) are annotated as protein with RHO_alpha_C_DMO-like (cd08878) domain, which was also considered as a rule to identify a ligX. For identification of other enzymes, local databases were setup and BLASTP was carried out.

Identification of plant secondary metabolites (PSMs)-degrading enzymes

Plant secondary metabolites (PSMs) and other xenobiotics are toxic to insects and their gut microbes, and gut microbiota may reduce PSM toxicity to the host[8]. PSMs can be divided into three major groups: phenolic acids, terpenoids and steroids, Nitrogen-containing alkaloids and sulphur-containing compounds[14]. High levels of non-protein

amino acids have been identified in certain plant families and have direct toxic effects on insects via several mechanisms [10].

The metabolic excretion of PSMs and other xenobiotics by insects tends to occur via insect-derived enzymes such as cytochrome P450s, glutathione-S-transferases (GSTs), carboxylesterases (COEs), and UDP-glucuronlytransferases (UGTs). And in the end, conjugated compounds are exported out of the cell by employing ATP-binding cassette (ABC) and other transmembrane transporters (for review, see[1]). Gut prophenoloxidase also detoxifies plant phenolics[20]. The enzymes catalyzing the isomerization of flavanones (chalcone isomerise (EC:5.5.1.6)), the reduction of chalcones (enoate reductase (EC:1.3.1.31)) and the cleavage of dihydrochalcones (phloretin hydrolase (EC:3.7.1.4))[2]. Tannin acyl hydrolase is an enzyme that hydrolyses the ester bonds of tannic acid to produce gallic acid and glucose and galloyl esters. Tannase (EC: 3.1.1.20) as inducible enzyme is produced by variety of microorganism such as bacteria, fungi and yeast [6].

Microbe-derived p450s were identified by the EC numbers (http://www.icgeb.org/~p450srv/P450enzymes.html) which were converted to GO IDs. Since PSMs consist of a wide range of compounds and their degradation occurs via a large number of metabolic pathways, it is difficult to collect all known degrading enzymes. We used the GO IDs (GO:0019748, GO:0009820, GO:0006721, GO:0009812, GO:0009698 and GO:0030638) and their children IDs to collect the enzymes involved in PSMs degradation. Enzymes involved in the degradation of geraniol, limonene and pinene were identified by KEGG annotation. The untargeted enzymes were removed manually based on the annotations from UniprotKB.

The known gut microbial enzymes with relevance to polyphenol metabolism are Quercetin 2,3-dioxygenase (Flavonol 2,4-dioxygenase, Quercetinase), dioxygenases, NADPH-dependent curcumin/dihydrocurcumin reductase (CurA), Daidzein reductase, Dihydrodaidzein reductase, and Tetrahydrodaidzein reductase [17]. We used Uniprot IDs presented in ref. [17] to collect these enzymes, and for those do not have GO annotations, local BLASTP was carried out.

As the url http://www.icgeb.org/~p450srv/P450enzymes.html does not work now, we presented ECs of P450 in the following Table S1.

Table S1 EC numbers of P450 used in this study.

EC	Recommended name	Family(gene)
1.3.3.9	secologanin synthase	CYP72A1
1.14.13.11	trans-cinnamate 4-monooxygenase	CYP73
1.14.13.12	benzoate 4-monooxygenase	CYP53
1.14.13.13	calcidiol 1-monooxygenase	CYP27
1.14.13.15	cholestanetriol 26-monooxygenase	CYP27
1.14.13.17	cholesterol 7-monooxygenase	CYP7
1.14.13.21	flavonoid 3'-monooxygenase	CYP75
1.14.13.28	3,9-dihydroxypterocarpan 6a-monooxygenase	CYP93A1
1.14.13.30	leukotriene-B4 20-monooxygenase	CYP4F
1.14.13.37	methyltetrahydroprotoberberine 14-monooxygenase	CYP93A1
1.14.13.41	tyrosine N-monooxygenase	CYP79
1.14.13.42	hydroxyphenylacetonitrile 2-monooxygenase	-
1.14.13.47	(-)-limonene 3-monooxygenase	-
1.14.13.48	(-)-limonene 6-monooxygenase	-
1.14.13.49	(-)-limonene 7-monooxygenase	-
1.14.13.52	isoflavone 3'-hydroxylase	-
1.14.13.53	isoflavone 2'-hydroxylase	-
1.14.13.55	protopine 6-monooxygenase	-
1.14.13.56	dihydrosanguinarine 10-monooxygenase	-
1.14.13.57	dihydrochelirubine 12-monooxygenase	-
1.14.13.60	27-hydroxycholesterol 7-monooxygenase	-
1.14.13.70	sterol 14-demethylase	CYP51
1.14.13.71	N-methylcoclaurine 3'-monooxygenase	CYP80B1
1.14.13.73	tabersonine 16-hydroxylase	CYP71D12
1.14.13.74	7-deoxyloganin 7-hydroxylase	-
1.14.13.75	vinorine hydroxylase	-
1.14.13.76	taxane 10-hydroxylase	CYP725A1
1.14.13.77	taxane 13-hydroxylase	CYP725A2
1.14.13.78	ent-kaurene oxidase	CYP701
1.14.13.79	ent-kaurenoic acid oxidase	CYP88A

1.14.14.1	unspecific monooxygenase	multiple
1.14.15.1	camphor 5-monooxygenase	CYP101
1.14.15.3	alkane 1-monooxygenase	CYP4A
1.14.15.4	steroid 11-monooxygenase	CYP11B
1.14.15.5	corticosterone 18-monooxygenase	CYP11B
1.14.15.6	cholesterol monooxygenase (side-chain-cleaving)	CYP11A
1.14.21.1	(S)-stylopine synthase	-
1.14.21.2	(S)-cheilanthifoline synthase	-
1.14.21.3	berbamunine synthase	CYP80
1.14.21.4	salutaridine synthase	-
1.14.21.5	(S)-canadine synthase	-
1.14.99.9	steroid 17-monooxygenase	CYP17
1.14.99.10	steroid 21-monooxygenase	CYP21
1.14.99.22	ecdysone 20-monooxygenase	-
1.14.99.28	linalool 8-monooxygenase	CYP111
4.2.1.92	hydroperoxide dehydratase	CYP74
5.3.99.4	prostaglandin-I synthase	CYP8
5.3.99.5	thromboxane-A synthase	CYP5

Reference List

1. Bansal R, Mian MAR, Mittapalli O, Michel AP. RNA-Seq reveals a xenobiotic stress response in the soybean aphid, Aphis glycines, when fed aphid-resistant soybean. BMC Genomics. 2014;15:1-14.

2. Braune A, Blaut M. Bacterial species involved in the conversion of dietary flavonoids in the human gut. Gut Microbes. 2016;7:216-34.

3. Ceballos SJ, Yu C, Claypool JT, Singer SW, Simmons BA, Thelen MP, Simmons CW, VanderGheynst JS. Development and characterization of a thermophilic, lignin degrading microbiota. Process Biochem. 2017;63:193-203.

4. de Gonzalo G, Colpa DI, Habib MHM, Fraaije MW. Bacterial enzymes involved in lignin degradation. J Biotech. 2016;236:110-9.

5. Dimarogona M, Topakas E, Christakopoulos P. Cellulose degradation by oxidative enzymes. Comput Struc t Biotechnol J. 2012;2:1-8.

6. Govindarajan RK, Revathi S, Rameshkumar N, Krishnan M, Kayalvizhi N. Microbial tannase: Current perspectives and biotechnological advances. Biocatal Agric Biotech. 2016;6:168-75.

7. Habrylo O, Evangelista DE, Castilho PV, Pelloux J, Henrique-Silva Fv. The pectinases from Sphenophorus levis: Potential for biotechnological applications. Int J Biol Macromol. 2018;112:499-508.

8. Hammer TJ, Bowers MD. Gut microbes may facilitate insect herbivory of chemically defended plants. Oecologia. 2015;179:1-14.

9. Hatakka A. Biodegradation of Lignin. In: Alexander Steinbuchel ed. Biopolymers Online. Wiley-VCH Verlag GmbH & Co. KGaA. 2005: 129-145

10. Huang T, Jander G, de Vos M. Non-protein amino acids in plant defense against insect herbivores: Representative cases and opportunities for further functional analysis. Phytochemistry. 2011;72:1531-7.

11. Kumar M, Verma S, Gazara RK, Kumar M, Pandey A, Verma PK, Thakur IS. Genomic and proteomic analysis of lignin degrading and polyhydroxyalkanoate accumulating β-proteobacterium *Pandoraea* sp. ISTKB. Biotechnol Biofuels. 2018;11:154.

12. Marchler-Bauer A, Bo Y, Han L, He J, Lanczycki CJ, Lu S, Chitsaz F, Derbyshire MK, Geer RC, Gonzales NR, Gwadz M, Hurwitz DI, Lu F, Marchler GH, Song JS, Thanki N, Wang Z, Yamashita RA, Zhang D, Zheng C, Geer LY, Bryant SH. CDD/SPARCLE: functional classification of proteins via subfamily domain architectures. Nucleic Acids Res. 2017;2016/11/28:D200-D203.

13. Moreira LRS, Filho EXF. Insights into the mechanism of enzymatic hydrolysis of xylan. Appl Microbiol Biot. 2016;100:5205-14.

14. Musilova L, Ridl J, Polivkova M, Macek T, Uhlik O. Effects of secondary plant metabolites on microbial populations: changes in community structure and metabolic activity in contaminated environments. Int J Mol Sci. 2016;17:1205.

15. Rahman AKM, Sugitani N, Hatsu M, Takamizawa K. A role of xylanase, α-L-arabinofuranosidase, and xylosidase in xylan degradation. Can J Microbiol. 2003;49:58-64.

16. Sainsbury PD, Mineyeva Y, Mycroft Z, Bugg TDH. Chemical intervention in bacterial lignin degradation pathways: Development of selective inhibitors for intradiol and extradiol catechol dioxygenases. Bioorg Chem. 2015;60:102-9.

17. Stevens JF, Maier CS. The chemistry of gut microbial metabolism of polyphenols. Phytochem Rev. 2016;15:425-44.

18. Tereshchenkova VF, Goptar IA, Kulemzina IA, Zhuzhikov DP, Serebryakova MV, Belozersky MA, Dunaevsky YE, Oppert B, Filippova IY, Elpidina EN. Dipeptidyl peptidase 4- An important digestive peptidase in *Tenebrio molitor* larvae. Insect Biochem Mol Biol. 2016;76:38-48.

19. Wefers D, Dong J, Abdel-Hamid AM, Paul HMl, Pereira GV, Han Y, Dodd D, Baskaran R, Mayer B, Mackie RI, Cann I. Enzymatic mechanism for arabinan degradation and transport in the thermophilic bacterium *Caldanaerobius polysaccharolyticus*. Appl Environ Microbiol. 2017;83:e00794-17.

20. Wu K, Zhang J, Zhang Q, Zhu S, Shao Q, Clark KD, Liu Y, Ling E. Plant phenolics are detoxified by prophenoloxidase in the insect gut. Sci Rep. 2015;5:16823.

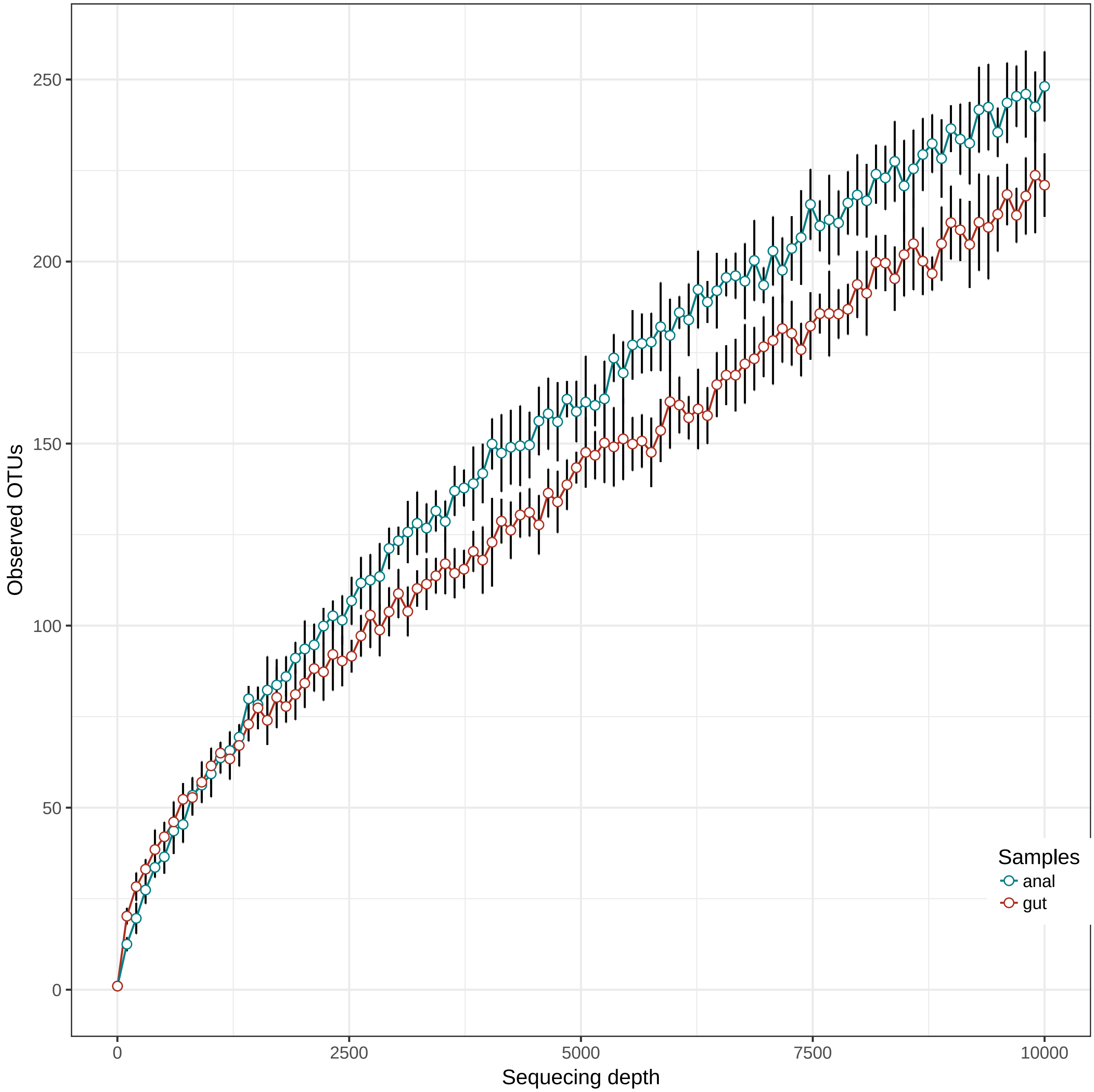

Fig. S1 Rarefaction curves.

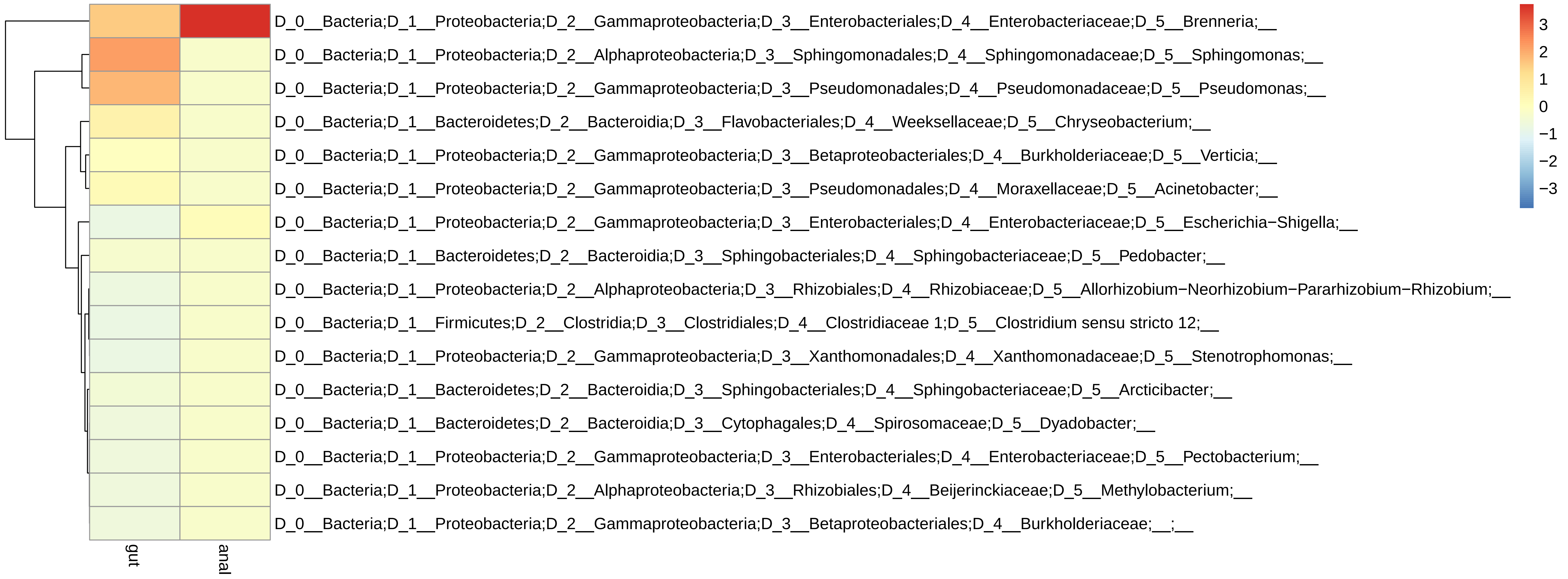

Fig. S2 A heatmap of bacteria identified from guts and anal droplets. Only those with frequency of more than 500 are shown

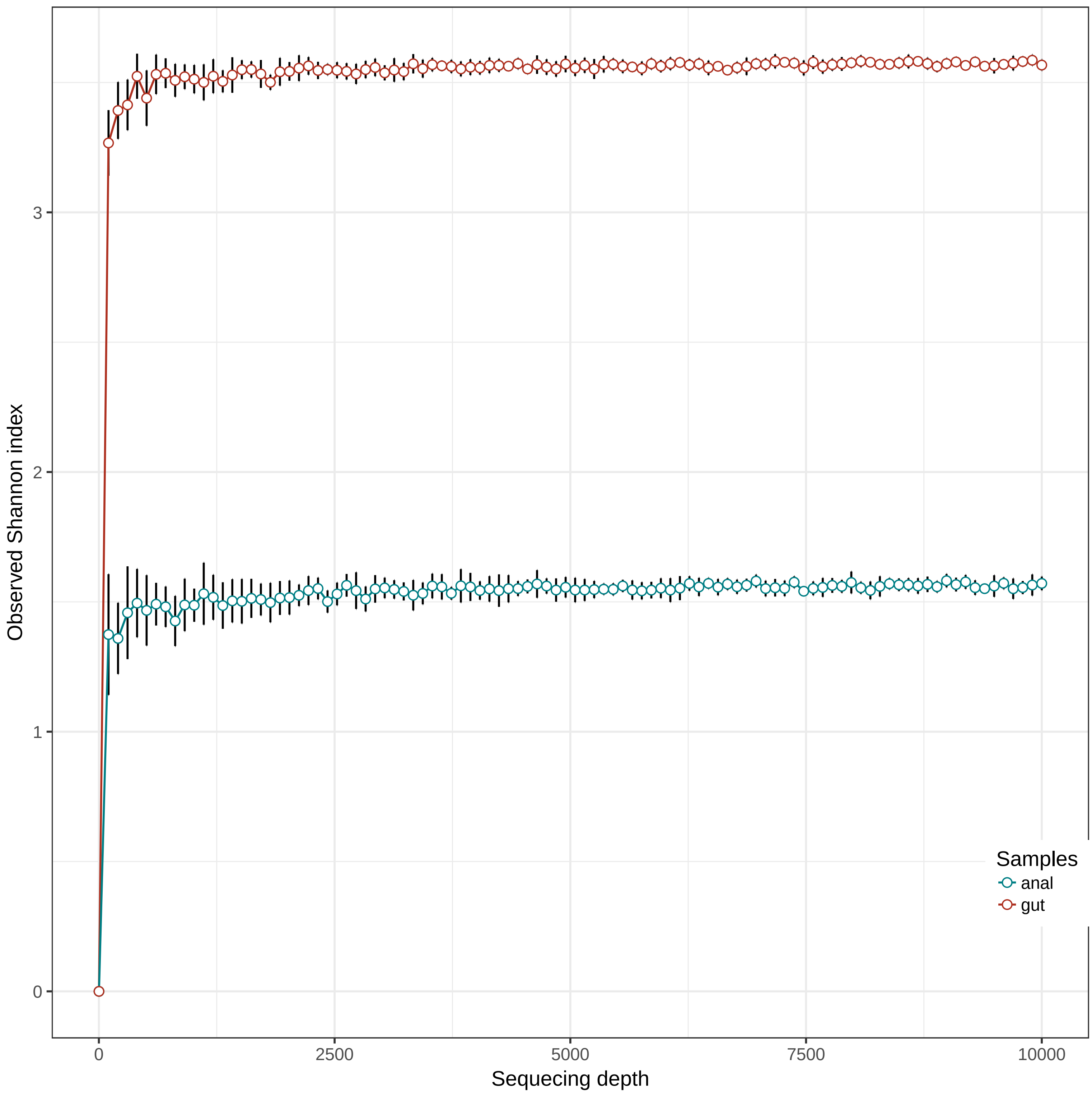

Fig. S3 Species diversity of bacterial community from gut and anal droplet

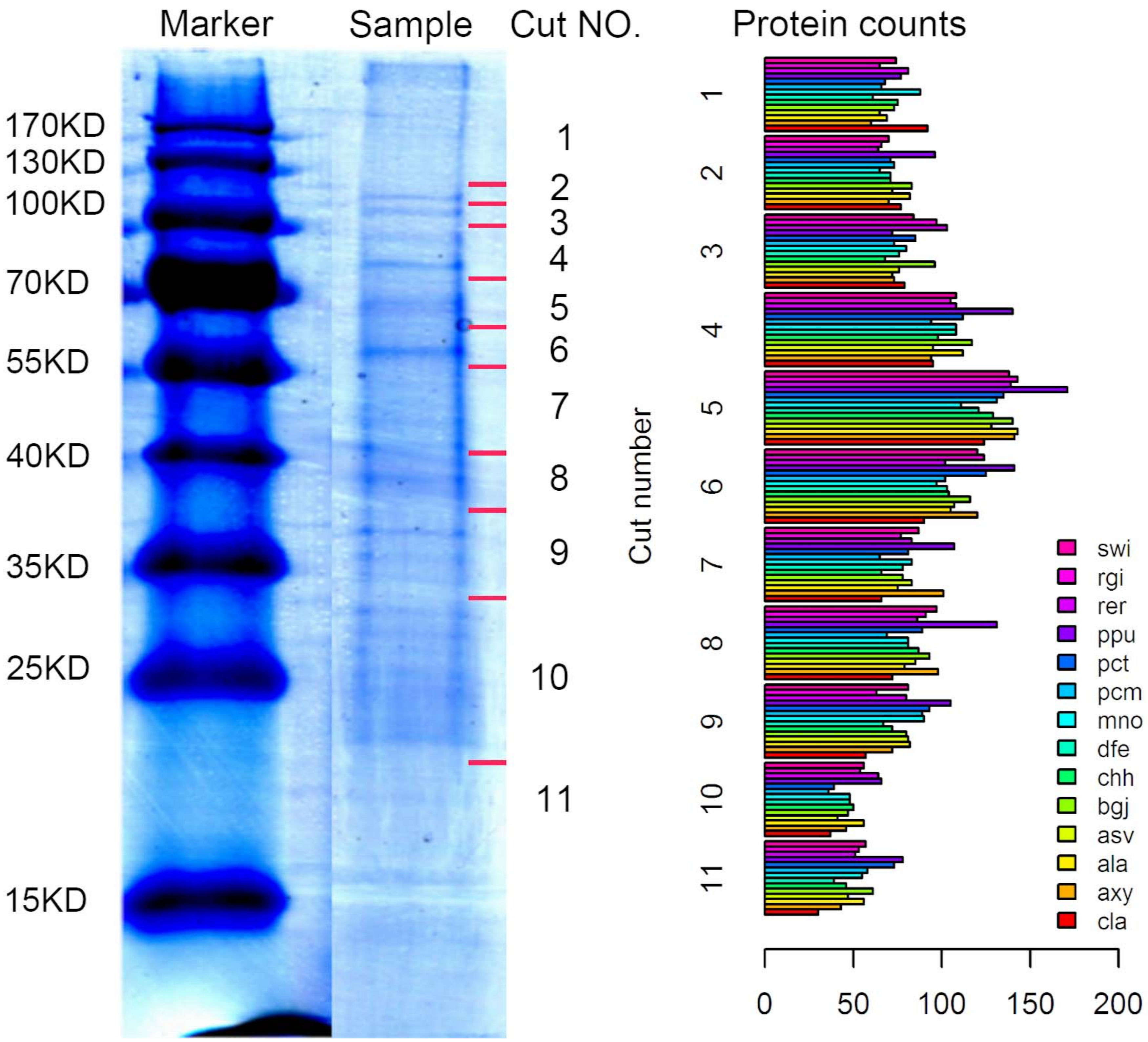

Fig. S4 SDS-PAGE of the anal droplets. The gel was cut into 11 segments at the red line for Q-TOF (Jing et al, 2018). The counts of identified protein from each segment are shown in a barplot.

Jing T, Wang F, Qi F, Wang Z. Insect anal droplets contain diverse proteins related to gut homeostasis. BMC Genomics. 2018; 19:784

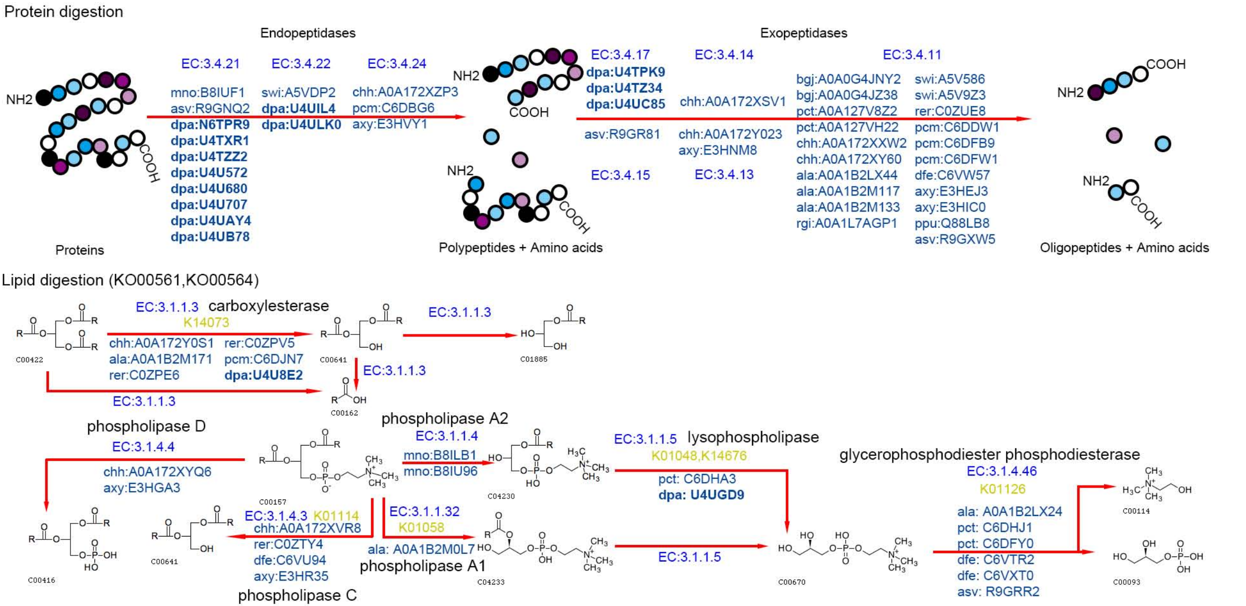

Fig. S5 Droplet enzymes digesting proteins and lipids. The digestion pathway of phosphatidylethanolamine was not shown, which is the same as that of phosphatidylcholine.

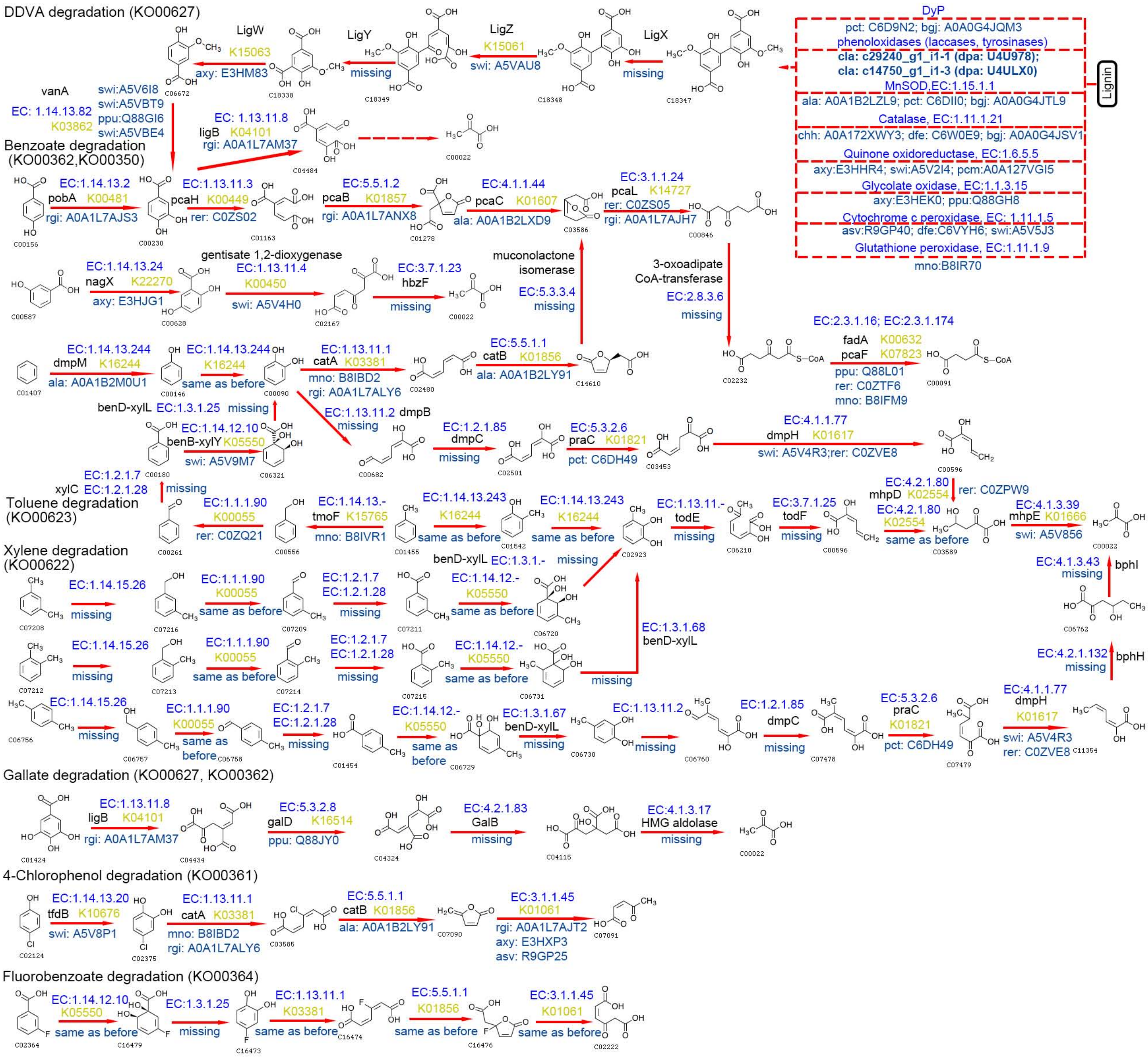

Fig. S6 Droplet enzymes degrading phonics.

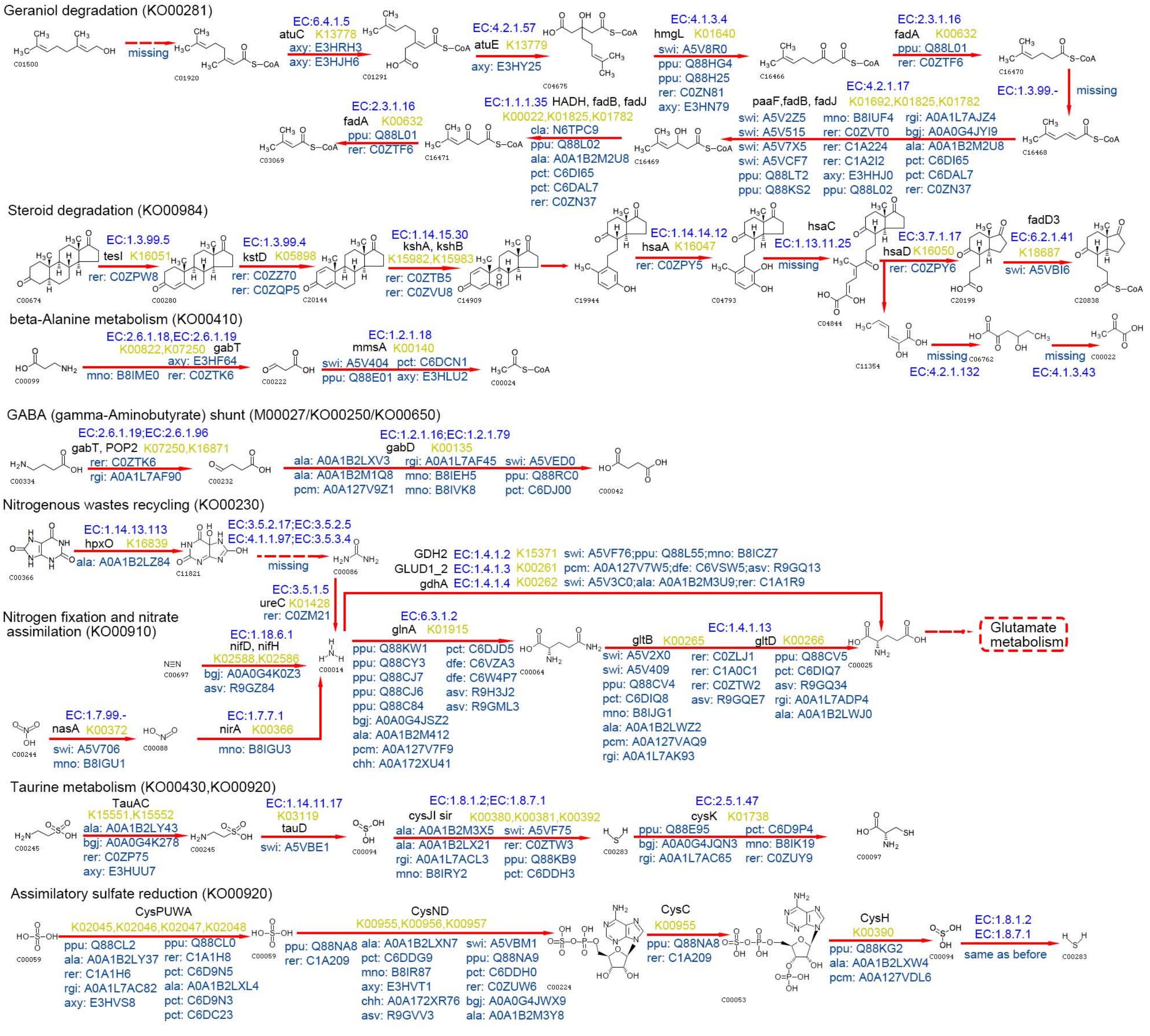

Fig. S7 Droplet enzymes degrading terpenoids, steroids, non-protein amino acids and involved in metabolism of nitrogen and sulfates.

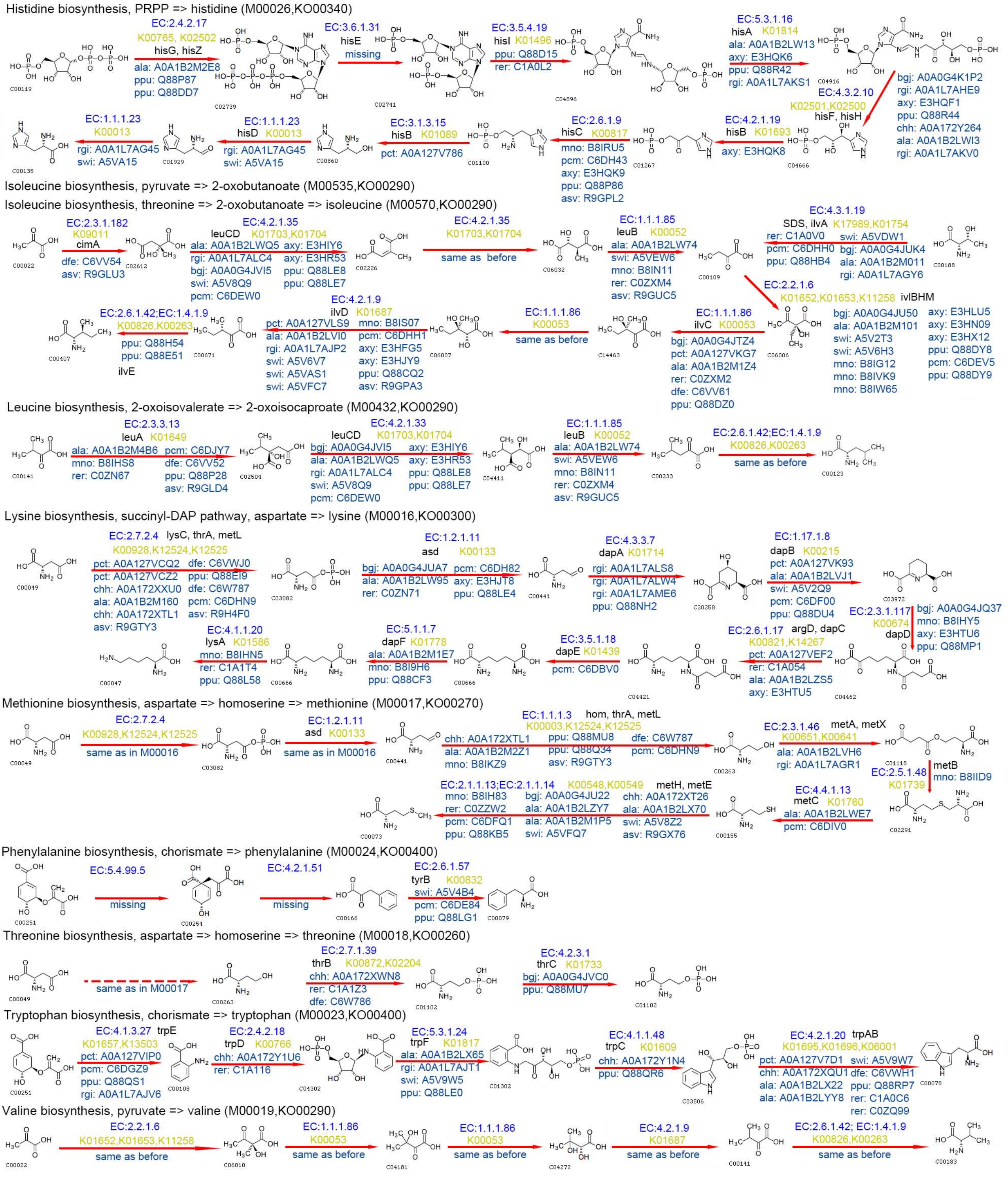

Fig. S8 Droplet enzymes involved in the biosynthesis of essential amino acids.

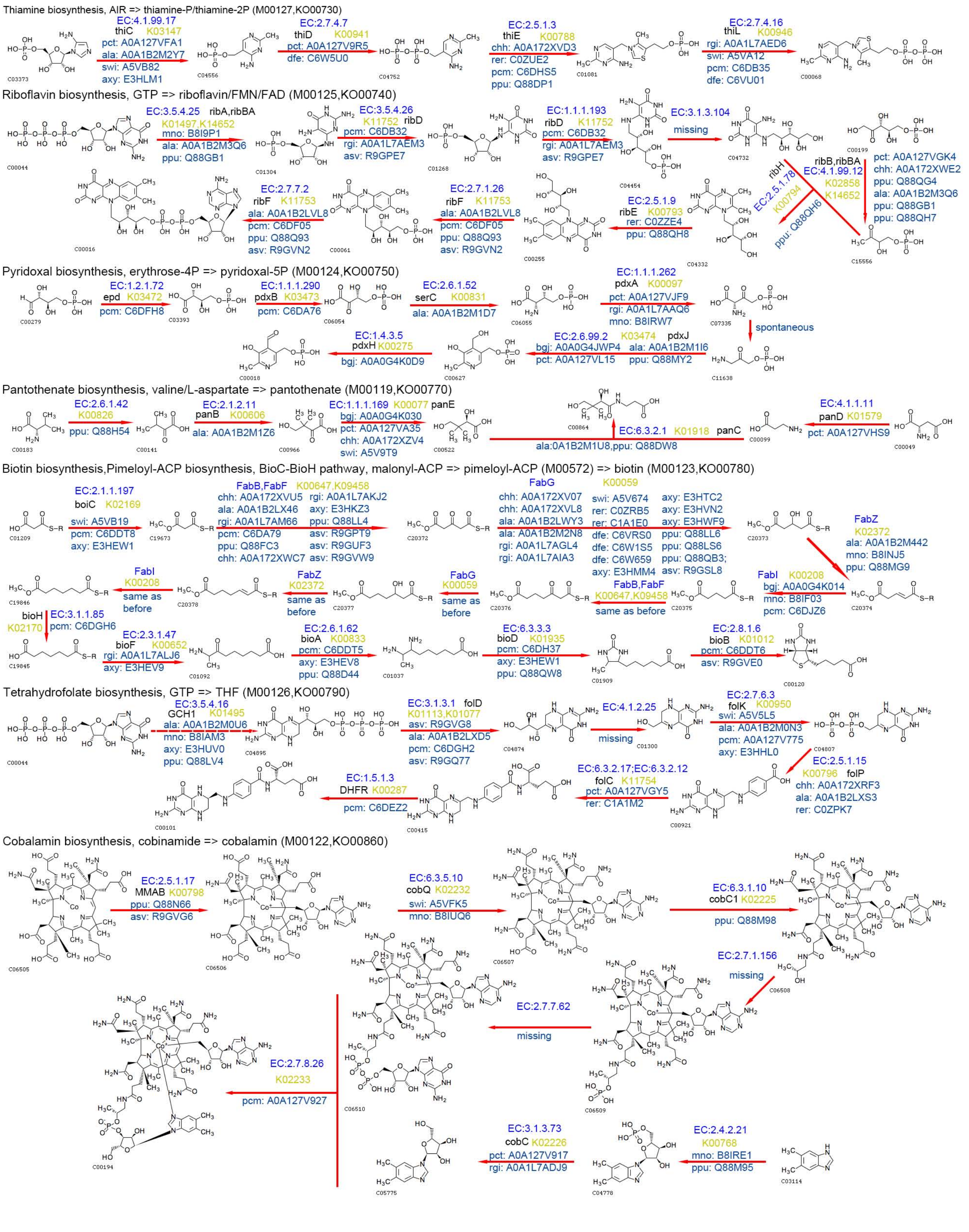

Fig. S9 Droplet enzymes involved in the biosynthesis of vitamins and cofactors.
